# Supplementary material for: A multi-adenylate cyclase regulator at the flagellar tip controls African trypanosome transmission
Source: Nat Commun. 2022 Sep 16;13:5445. doi: 10.1038/s41467-022-33108-z (PMC9481589; doi:10.1038/s41467-022-33108-z)
Supplement: Supplementary file 1 — Supplementary Information [file 41467_2022_33108_MOESM1_ESM.pdf]

Supplementary Information

Bachmaier et al., A multi-adenylate cyclase regulator at the flagellar tip controls African trypanosome transmission

Supplementary Figure 1

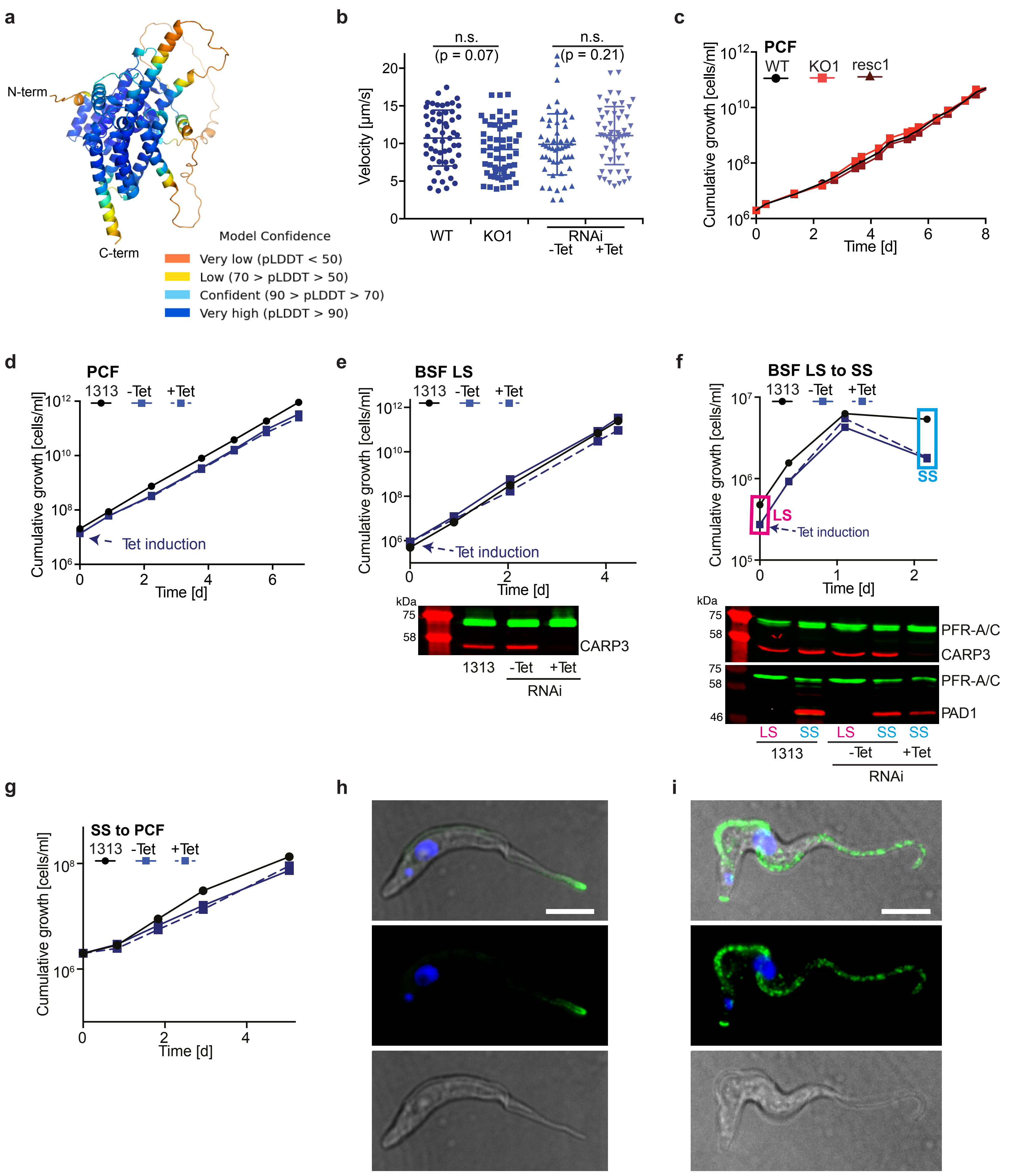

CARP3 is not essential for growth or differentiation of *T. brucei* but shows life cycle stage-specific localization

(a) Cartoon representation of a predicted model of *T. brucei* CARP3 using AlphaFold[37]. Model confidence is illustrated using the predicted local-distance difference test (pLDDT) score, indicated by the color-coding.

(b) Analysis of single cell mean velocity (mean ± SD; n = 56 (WT), 57 (KO1), 53 (RNAi -Tet), 54 (RNAi +Tet)) of procyclic form (PCF) AnTat 1.1 WT, *carp3* KO and RNAi cell lines. n.s.: non-significant ($p > 0.05$; One-Way ANOVA corrected by Šidák's multiple comparisons test; adjusted p values are given in the figure).

(c) Representative growth curves of PCF AnTat 1.1 WT, *carp3* KO (KO1) and *CARP3* rescue (resc1) cell lines. Corresponding Western blot is shown in Fig. 1a.

(d, e) Representative growth curves of PCF (**d**) or bloodstream form (BSF) (**e**) AnTat 1.1 1313 and tetracycline-inducible *CARP3* RNAi cell lines. RNAi was induced by addition of 5 µg/mL tetracycline (+Tet condition). Repression of CARP3 levels was confirmed by Western blot analysis (e) with PFR-A/C as loading control. Western blot corresponding to (d) is shown in Fig. 1b.

(f, g) Growth of cell lines as in (d, e) during differentiation from BSF long slender (LS) to short stumpy (SS) (**f**) or during SS to PCF differentiation (**g**). Western blot in (f) shows expression of CARP3 and the stumpy marker protein PAD1. PFR-A/C serves as loading control. The growth curve in (f) was started at time point 0 h with long slender cells at a density of 5-8 × 10^5 cells/mL and Tet induction of *CARP3* RNAi. Growth was monitored over 52 h without culture dilution, resulting in development into PAD1-expressing short stumpy forms. The growth curve in (g) was initiated with the SS cells from (f).

(h, i) Indirect immunofluorescence analysis of CARP3 (green) in *T. brucei* AnTat 1.1 procyclic forms (**h**) or long slender bloodstream forms (**i**). DNA was stained with DAPI (blue). Fluorescence channels were merged with the differential interference contrast (DIC). Scale bars 5 µm.

Supplementary Figure 2

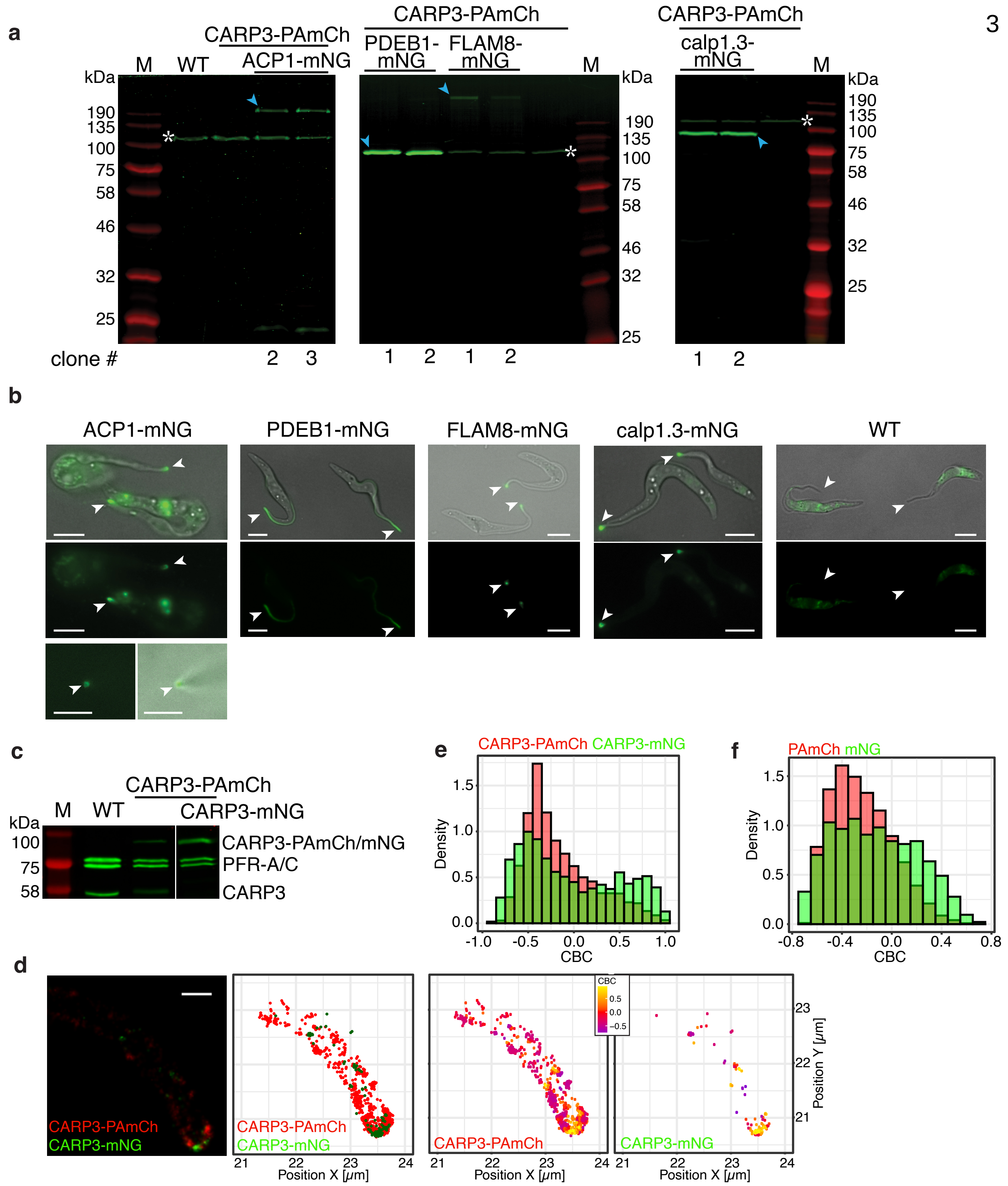

Generation of trypanosome cell lines for photoactivated localization microscopy of CARP3 and putative colocalization partners.

(a) In-gel fluorescence and **(b)** live cell fluorescence microscopy of procyclic *T. brucei* AnTat 1.1E expressing CARP3-PA-mCherry (PAmCh) and ACP1P, PDEB1, FLAM8, or calpain 1.3 (calp1.3), respectively, C-terminally fused to mNeonGreen (mNG) (labeled by blue arrowheads in (a)). Wild type (WT) cells were included as control. The white asterisk (*) marks an endogenous, autofluorescent protein that we identified as fumarate reductase[124]. Note that the PDEB1-mNG fusion protein runs at the same apparent molecular weight as the autofluorescent fumarate reductase. White arrowheads in (b) point towards flagellar tips. The second image shown for ACP1-mNG displays a fluorescent flagellar tip from the top perspective. Scale bars 5 μm. M protein molecular weight marker.

(c) Western blot analysis (anti-CARP3, anti-PFR-A/C (loading control)), **(d)** PALM imaging and **(e)** colocalization analysis (as in Fig. 2) of procyclic *T. brucei* AnTat 1.1E expressing CARP3-PAmCherry (PAmCh, red) and CARP3-mNeonGreen (mNG, green). While WT cells express CARP3 (~57 kDa) from two endogenous alleles (c, lane 'WT'), the hemizygous *in situ* CARP3-PAmCherry cells (middle lane in (c)) express CARP3 from one wild type allele (~57 kDa) and one endogenous CARP3-PAmCherry fusion (~85 kDa). The CARP3-PAmCherry/CARP3-mNG (c, right lane) cell line expresses two endogenously tagged CARP3 alleles, both resulting in proteins with similar molecular weight (~85 kDa), one fused to PAmCherry, the other fused to mNG, resulting in replacement of both endogenous alleles. Scale bar in (d) 0.5 μm.

(f) CBC values distributions for two simulated independent Poisson point patterns characterized by densities equal to the ones from procyclic *T. brucei* AnTat 1.1E expressing CARP3-PAmCherry and CARP3-mNeonGreen. The point patterns are confined within a rectangular area of 1 μm width (approximation of a straight flagellum).

Supplementary Figure 3

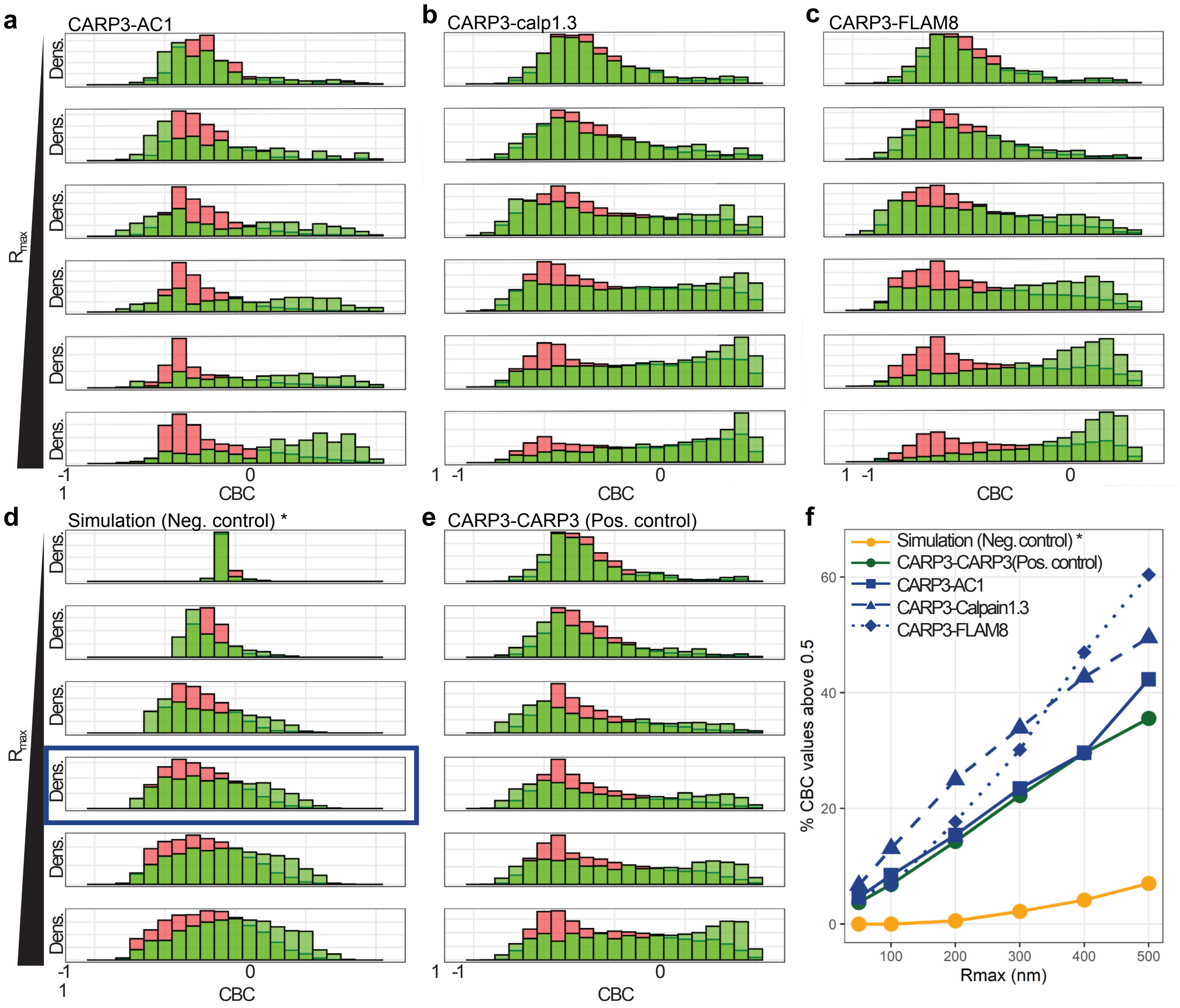

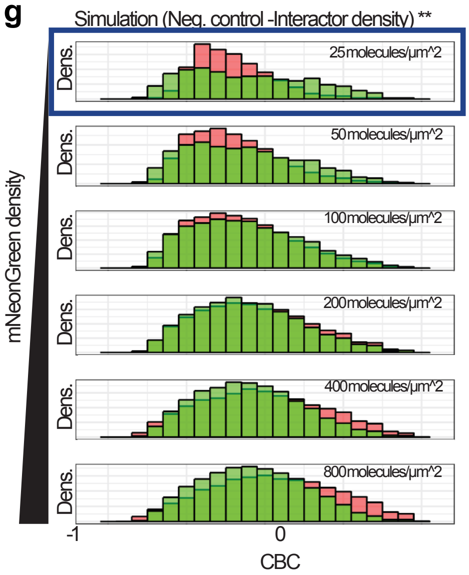

h

Density mNeonGreen (molecules/μm^2)	Density PAmCherry (molecules/μm^2)	mNeon CBC>=0.5 (percentage)	PAmCherry CBC>=0.5 (percentage)
25	134.8	5.07	1.32
50	134.8	7.32	2.03
100	134.8	5.55	3.97
200	134.8	5.42	7.23
400	134.8	5.30	10.20
800	134.8	4.77	15.09

* CBC values for biological samples and simulations with increasing R_{max} were determined at 5 nm intervals in order to guarantee a minimum of 10 intervals also at the smallest R_{max}. The concentration of PAmCherry and mNeonGreen is derived from the positive control.

** CBC values from simulations with increasing mNeonGreen densities were determined at 10 nm intervals to fasten the computational process. Given that all putative interactors are tested against CARP3, the density of PAmCherry molecules is not expected to change significantly between samples. The increase in mNeonGreen density is not reflected by an increase in CBC values for the mNeonGreen molecules, but only for the PAmCherry molecules. It is therefore not necessary to run a separate simulation for each tested CARP3-interactor pair.

Coordinate-based colocalization (CBC) analysis of PALM data for CARP3 and putative colocalization partners.
(a-e) CBC value density distributions for CARP3-AC1 (**a**), CARP3-calpain 1.3 (**b**), CARP3-FLAM8 (**c**), negative control (**d**) and CARP3-CARP3 (**e**) calculated for six different R_{max} (50, 100, 200, 300, 400, 500 nm). The CBC value density distributions for mNeonGreen-tagged proteins relative to PAmCherry-tagged proteins is shown in green, while the opposite is shown in red. Interval width = 5 nm.
(f) The percentage of CBC values above or equal to 0.5 relative to R_{max} derived from (a-e) is plotted for each fluorescent protein pair and compared to the positive and the negative control.
(g, h) CBC value density distributions calculated for simulations characterized by increasing mNeonGreen concentrations (25, 50, 100, 200, 400 and 800 molecules/μ^2) and constant PAmCherry concentration (**g**). R_{max} = 300 nm, interval width = 10 nm. The percentage of CBC values above or equal to 0.5 (mNeonGreen to PAmCherry and vice versa) was determined and compared for all mNeonGreen densities to evaluate the necessity of separate negative controls for each protein pair (**h**).

Supplementary Figure 4

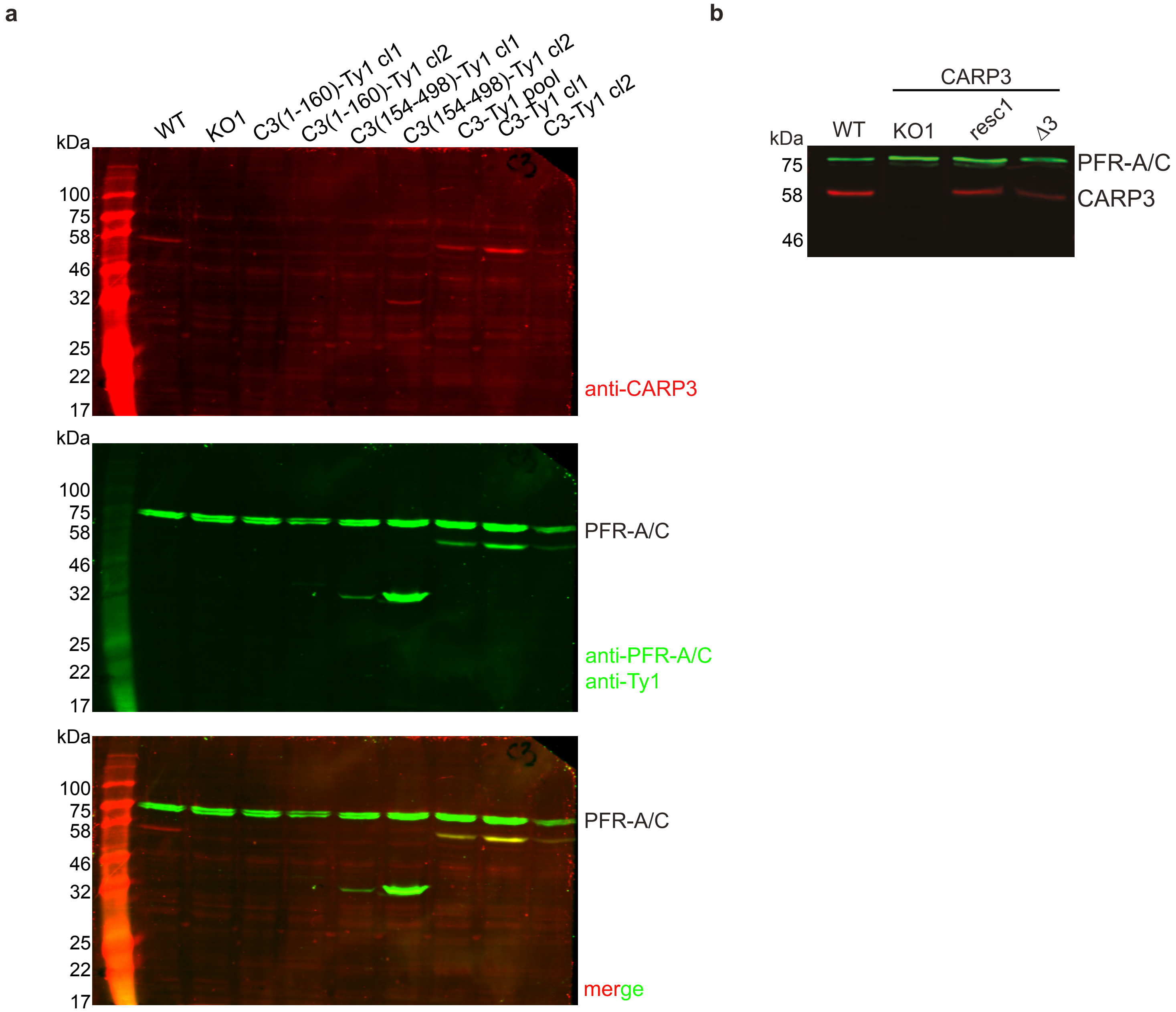

Expression of CARP3 mutant proteins

(a) Western blot analysis of procyclic *T. brucei* AnTat 1.1 constitutively overexpressing CARP3-Ty1 (pool and clones 1 and 2), CARP3(1-160)-Ty1 (clones 1 and 2) or CARP3(154-498)-Ty1 (clones 1 and 2) or **(b)** *in situ* rescue with full-length *CARP3* (resc1) or *CARP3Δ3* (Δ3) in a *carp3* knock out (KO) background. Wild type (WT) and KO (KO1) cell lines were included as controls. Western blots were probed with anti-CARP3, anti-Ty1 (only in (a)) and anti-PFR- A/C (loading control).
Note that CARP3(1-160)-Ty1 (calculated molecular weight 19.5 kDa) is not detectable by Western blot but only by immunofluorescence microscopy (see Fig. 2k). M: protein molecular weight marker.

Supplementary Figure 5

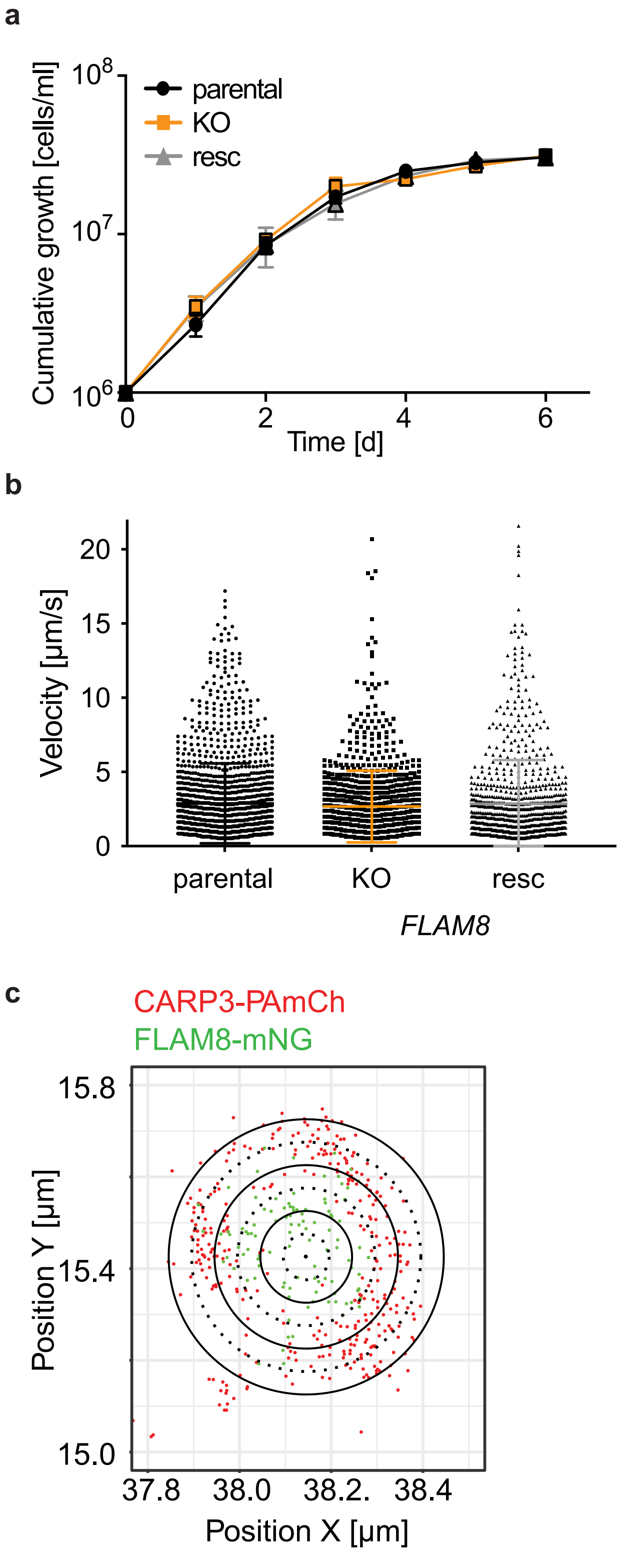

***FLAM8* knock out cells have no growth or motility phenotype**
(a, **b)** Representative growth curves **(a)** and analysis of single cell mean velocity **(b)** of AnTat 1.1E 'Paris' parental, *flam8* knock out (KO) or *FLAM8* rescue (resc) expressing the red triple marker. (b) shows mean ± SD of n = 1502 (parental); n = 898 (KO); n = 898 (resc). Note that the ~4-fold difference in the mean cell velocity between control cell lines in Supplementary Fig. 5b and Supplementary Fig. 1b is due to differences in cell densities, methyl cellulose concentrations and a different experimental set-up.
(c) Single molecule localization of CARP3-PAmCherry and FLAM8-mNG from Fig. 3e shown via centroids. Rings are drawn in 50 nm steps from center of flagellar tip cross-section. Dotted lines 50 nm, solid lines 100 nm.

Supplementary Figure 6

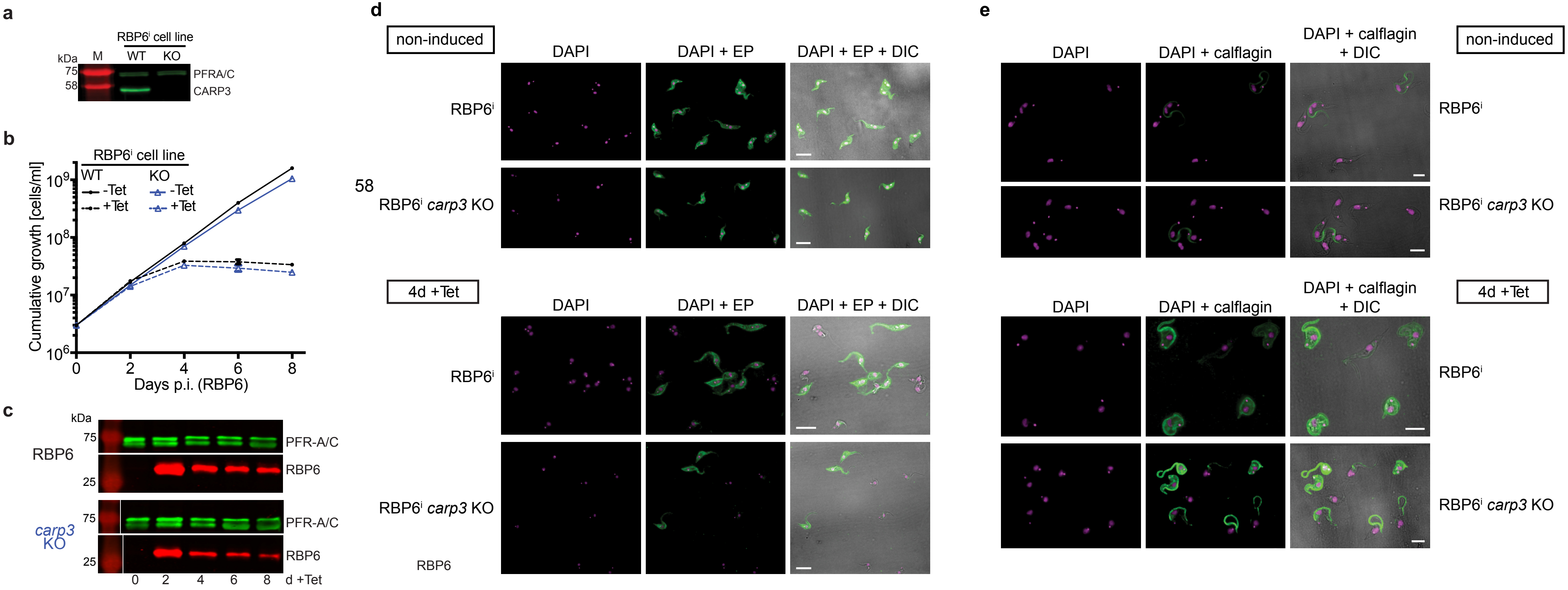

***CARP3* KO parasites are fully differentiation-competent in the *in vitro* RBP6 overexpression system**

(a) Western blot analysis of CARP3 expression in a homozygous deletion mutant of *carp3* (KO) generated in a procyclic *T. brucei* EATRO 1125 cell line allowing (Tet)-inducible RBP6 overexpression (RBP6ⁱ). PFR-A/C serves as loading control.

(b, **c)** Cumulative growth (**b**) and Western blot (**c**) analyses of cell lines as in (a) with or without Tet induction (10 µg/mL) of RBP6 overexpression. Growth curves are mean ± SD of n = 3 for +Tet; n = 1 for -Tet. Western blots show inducible overexpression of RBP6 for both cell lines. PFR-A/C serves as loading control.

(d, **e)** Indirect immunofluorescence analysis of EP procyclin (**d**) or calflagin (**e**) in the cell lines indicated. Marker protein expression was analyzed before (non-induced; upper panels) and four days after induction of RBP6 expression with 10 µg/mL tetracycline (4d +Tet; lower panels). DNA was stained with DAPI (magenta). Scale bars 5 µm.

Supplementary Figure 7

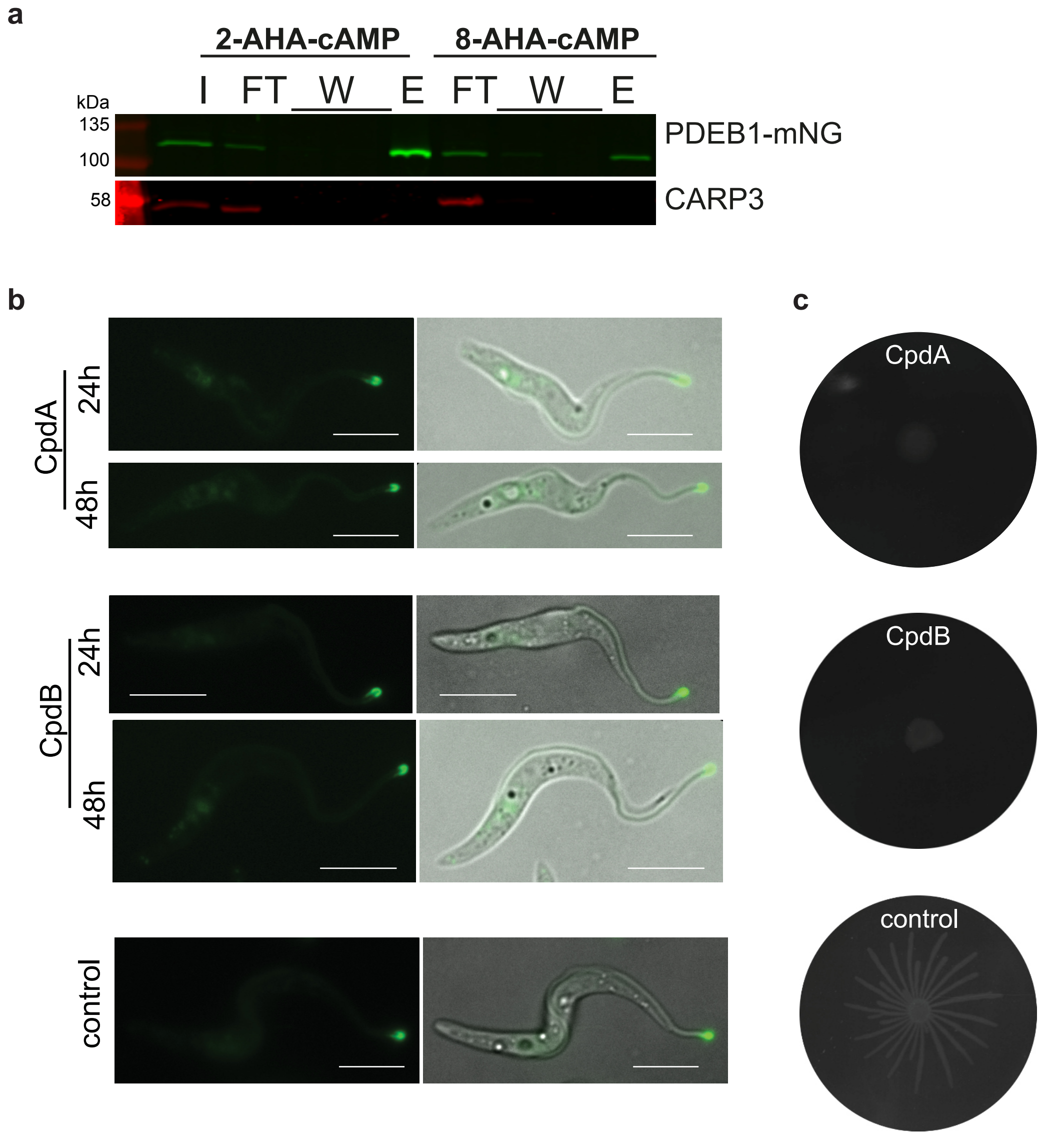

Expression or localization of CARP3 is not dependent on intracellular cAMP levels
(a) Pull-down assay using cAMP-coupled agarose beads (2-AHA or 8-AHA linker) and lysate of *T. brucei* AnTat 1.1E expressing PDEB1-mNeonGreen (PDEB1-mNG). PDEB1- mNG serves as positive control for cAMP binding and was detected by in-gel fluorescence; CARP3 was detected by immunoblotting using rabbit anti-CARP3. Lanes represent input material (I), flow-through (FT), washes (W) and eluted material (E).
(b) Fluorescence microscopy of CARP3-YFP (green) in procyclic *T. brucei* AnTat 1.1E in the presence (24h, 48h) or absence (control) of 1 µM CpdA or CpdB for 24 h or 48 h, respectively. Scale bars 5 µm.
(c) Social motility assay of CpdA- or CpdB-treated (1 µM) or untreated cells as in (b).

Supplementary Figure 8

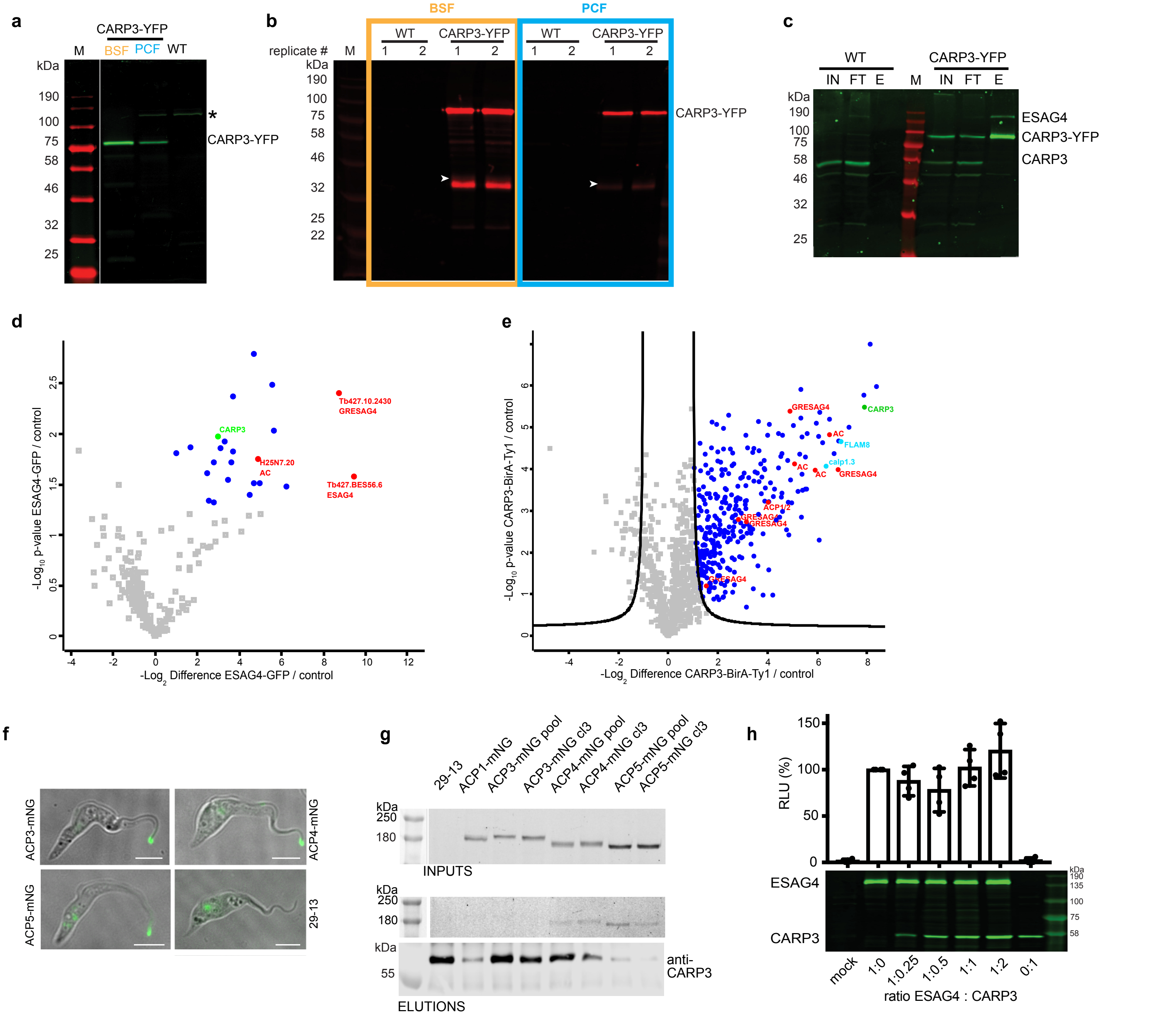

CARP3 interacts with ACs and regulates their abundance

(a) In-gel fluorescence of *T. brucei* BSFs and PCFs of strain AnTat 1.1E expressing CARP3-YFP *in situ*. Procyclic wild type cells (WT) were loaded as control. The asterisk (*) marks an autofluorescent fumarate reductase[124].

(b) GFP trap pull-down of cell lines from (a). The Western blot probed with anti-CARP3 shows the eluted fractions of two replicates each. The band at ~35 kDa (white arrowhead) is probably a proteolytic degradation product of CARP3.

(c) GFP trap pull-down in *T. brucei* MiTat 1.2 BSF wild type (WT) or CARP3-YFP cells. The Western blot was probed with anti-CARP3 and anti-ESAG4. IN input; FT flow-through; E elution.

(d) GFP trap pull-down in *T. brucei* MiTat 1.2 BSF 13-90 (control) or derived ESAG4-GFP expressing cells. The in-gel fluorescence analysis shows expression and solubility of ESAG4-GFP upon detergent lysis in the input fraction and pull-down of ESAG4-GFP in the eluted fraction. The Volcano plot displays proteins plotted according to p-value and fold change derived from mass spectrometry analysis of two replicate pull-downs. Significantly enriched proteins (ESAG4-GFP / control) (two-sided Student's t-test, p-value ≤ 0.05) are represented by blue dots. AC isoforms are shown in red, CARP3 in green.

(e) CARP3 proximity proteomics using BioID. Volcano plot representation of CARP3 BioID comparing pull-down of biotinylated proteins from BSF *T. brucei* Δ*carp3*/CARP3-BirA*-Ty1 versus Δ*carp3*/CARP3. Proteins are plotted according to p-value and fold change. Significantly enriched proteins (CARP3-BirA*-Ty1 / control) (two-sided Student's t-test, p-value ≤ 0.05, s_0=2) are represented by blue dots and localize above the significance line on the right. CARP3: green; FLAM8 and calpain 1.3: cyan; AC isoforms: red.

(f) Fluorescence microscopy of procyclic *T. brucei* 29-13 and derived ACP3-, ACP4- or ACP5-mNeonGreen expressing cells. Scale bars 5 µm.

(g) CARP3 IP in procyclic *T. brucei* 29-13 and derived ACP1-, ACP3-, ACP4- or ACP5-mNeonGreen expressing cell lines. Upper panel: in-gel fluorescence analysis of ACP-mNG in input fractions (INPUTS). Lower panels: in-gel fluorescence analysis of ACP-mNG and Western blot detection of CARP3 in eluted fractions (ELUTIONS).

(h) Dual-Luciferase® cAMP reporter assay in HEK cells transfected with ESAG4 and CARP3 expression vectors at the indicated ratios. Equal amounts of total DNA were transfected in all conditions. Relative light units (RLU) of the dual-luciferase assay were normalized to ESAG4 protein levels with one representative Western blot (anti-CARP3, anti-ESAG4) shown. The RLU value for the 1:0 ratio of ESAG4:CARP3 was set to 100%. The graph shows mean ± SD of four independent biological replicates.

Supplementary Figure 9

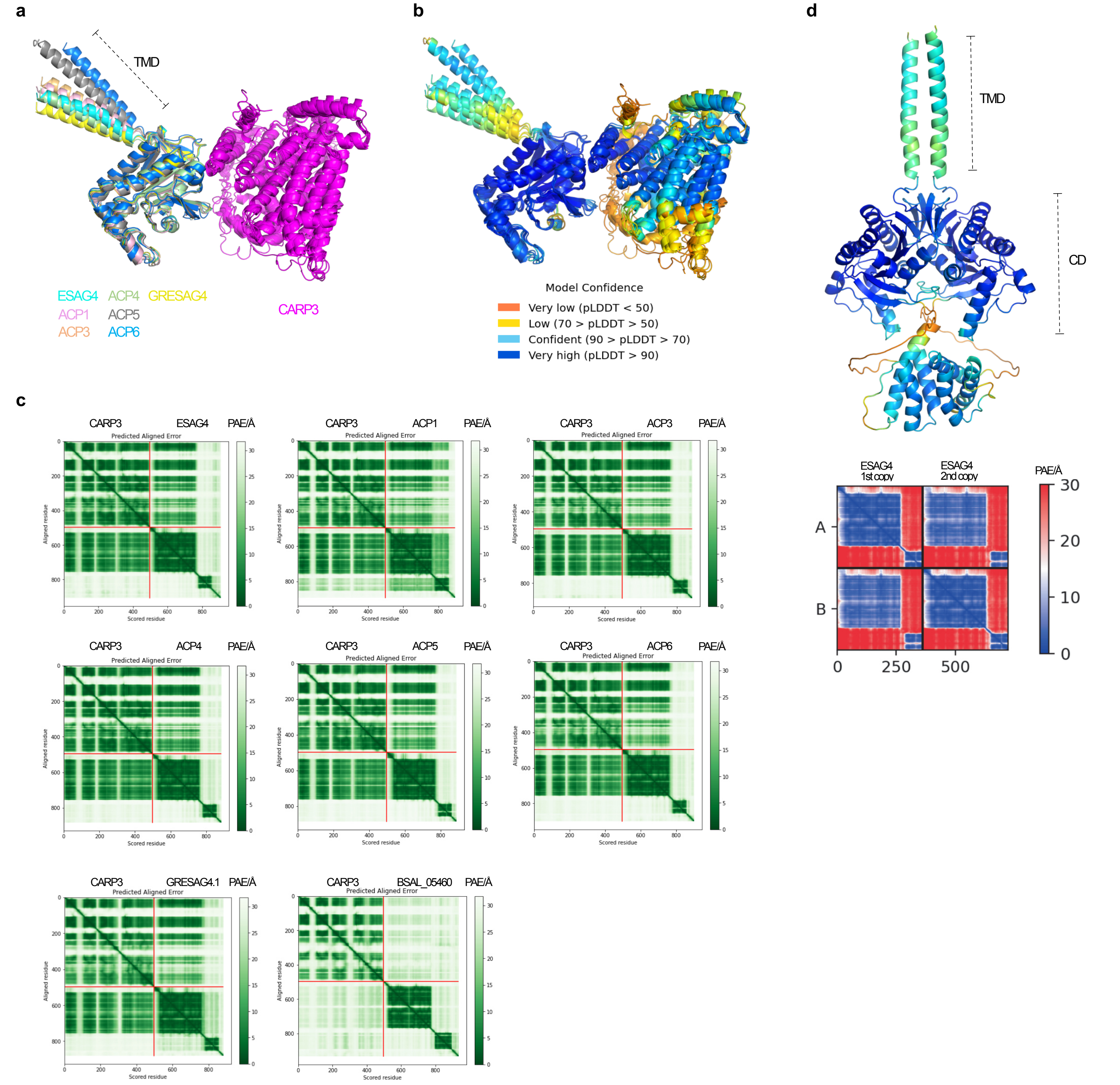

Structure modeling of CARP3-AC complexes using AlphaFold

(a) Cartoon representation of seven AlphaFold-generated models of CARP3 (magenta) in complex with the intracellular catalytic domain of different ACs (each AC isoform is shown by a different color, as indicated; ESAG4: Tb427.BES40.13; ACP1: Tb927.11.17040; ACP3: Tb927.7.7470;ACP4: Tb927.10.13040; ACP5: Tb927.11.13740; ACP6: Tb927.9.15660; GRESAG4.1: Tb927.6.760). The transmembrane domain (TMD) of the receptor-type ACs is labeled. The AC extracellular N-terminal part was not included in the structure prediction. The models are shown after superpositioning of CARP3.

(b) Same as panel (a) but colored according to local model confidence using the predicted local-distance difference test (pLDDT) score.

(c) Predicted alignment error (PAE) plots for the models shown in panels (a, b), indicating the confidence of the complex modelling. The PAE for the predicted model of *T. brucei* CARP3 in complex with a receptor-type transmembrane AC (BSAL_05460) from the distantly related kinetoplastid *Bodo saltans* that lacks a CARP3 orthologue was included as a negative control.

(d) AlphaFold-generated model for an ESAG4 AC homo-dimer (ESAG4 amino acids 862-end, lacking the extracellular N-terminal part) color-coded according to pLDDT score. TMD transmembrane domain; CD catalytic domain. Corresponding pymol states to (a) and (d) see Supplementary Data 6 and 7.

Supplementary Table 1. Summary of coordinate-based colocalization (CBC) analysis

Coordinate-based colocalization (CBC) analysis of CARP3-PAmCherry with different mNeonGreen fusion proteins.						
		Positive Control	Negative Control (simulation)			
		CARP3-CARP3	Poisson	CARP3-FLAM8	CARP3-Calpain	CARP3-AC1
**CARP3**	% CBC≥0.5 (PAmCherry)	8.47	0.24	12.84	21.32	2.52
**Interactor**	% CBC≥0.5 (mNeon)	22.22	2.19	30.14	33.90	23.46

Supplementary Table 2: List of primers used for cloning

Name	Sequence
CARP3_fw_HindIII	GTGCAAGCTTATGGGAGGAGGTTCATCC
CARP3_Ct_fw_HindIII	GTGCAAGCTTATGTCGCACACTCATG
CARP3_Ty1_Ct_rev_<br>BamHI	GTGCGGATCCTTAGTCAAGTGGGTCCTGGTTAGTATGGACCTCGTTCAATTGGT<br>CA
CARP3_Ty1_Nt_rev_<br>BamHI	GTGCGGATCCTTAGTCAAGTGGGTCCTGGTTAGTATGGACCTCCAAAAGCACA<br>TGA
PAmCherry BamHI<br>FWD	GAGCAGGGATCCATGGTGAGCAAGGGCGA
PAmCherry SacI REV	GGCGCCGAGCTCTTACTTGTACAGCTCGTCC
pPOTv4_Lr_mCherry_<br>TY_CARP3 fw	AACAGAGTCTCATGCGCCGTGTGTTGGAGACCCGTAAACGCGGTGGTGCTCTT<br>TTCA<br>TGATGTTTGATGACCAATTGAACGGATCGAGCGGTGGTGGCGG
pPOTv4_Lr_mCherry_<br>TY_CARP3 rev	CCTTGACGCTTCCATTTCTTTTTCCTTTTTTTCTTTTCTCACATCCACCCATCCTA<br>TTTT<br>CTTCTTTGCTTCCTTTGCATCCAATTTGAGAGACCTGTGC
CARP3_Δ3_HindIII_fw	ATCGATAAGCTTATGTCATCCGTTGAAGAC
ACP1orfF	GCATGGTACCACATGGCTGCCCGCACAGAG
ACP1orfR	GCATCTCGAGGTTTTCCTCCTTTGGGGTTGA
ACP1utrF	GCATGGATCCGGGTCGTAGGCATGCGCCAT
ACP1utrR	GCATTCTAGAGGGGGAGCGGGCGCCTCAAT
ACP6orfF	GCATGGTACCGAGGCATATGTGGCGGATG
ACP6orfR	GCATCTCGAGCTGCTTCCCCTTTTCCTCC
ACP6utrF_2	GCATGGATCCTAACAGCAGTAGTGAATTG
ACP6utrR_2	GCATTCTAGAACTTCACCTTCATCCTTCA
mNeon_XhoI_fw	GGTAATCTCGAGATGGTGAGCAAGGGC
mNeon_SalI_rev	GGCAGGGTCGACTTACTTGTACAGCTC
ACP3_ORF_FW	CTTGATGGTACCCGAACACGGCAGCAAGGACAGAGAG

ACP3_ORF_REV	CCAGATCTCGAGAAAATCACCCAGCTCCCCCACGTGATTGGAAA
ACP3_UTR_FW	GGCGCGGGATCCATTAGGATCAAAAGAAGTTA
ACP3_UTR_REV	GGCGATTCTAGACTGATATGGAGGTTCCGTTTTGGAT
ACP4_ORF_FW	CTTGATGGTACCTGCGCGGACGGAAAATGTGACGAAC
ACP4_ORF_REV	CCAGATCTCGAGAAACTTATCAAAATCCGTGGTCCGATTGGGG
ACP4_UTR_FW	CTTGATGGATCCGGGTTTTGGGGGGTTAATGGCACAA
ACP4_UTR_REV	GGCGATTCTAGAATTTCACCGCCGGAGACGTTGTTGA
ACP5_ORF_FW	CTTATGGTACCATGGCTGCGCGGACGGAGA
ACP5_ORF_REV	CTTATCTCGAGCCGCTGCGCTTCGGGGTTT
ACP5_UTR_FW	CCAATGGATCCAAACCACTCCACGAACTAATGACAGG
ACP5_UTR_REV	GGCATTCTAGAGAAGGAGTGTTCCCTGCGATAA
CARP3_hairpin_up_HindIII	GAGTAAGCTTTCAGAGGAATCAGGCGGAG
CARP3_hairpin_up_BamHI	GAGTGGATCCTCAGAGGAATCAGGCGGAG
CARP3_hairpin_sense_XhoI	CTGCCTCGAGCGTTTGCATTTCCATCATC
CARP3_hairpin_antisense_XhoI	GCCACTCGAGCGATTGTCCGACTTTCTTC
mNeon_BamHI_fw	TCTTATGGATCCGGTGGCGGAATGGTGAGCAAGGGCGAGG
mNeon_EcoRI_rev	GGAGGAGAATTCTTACTTGTACAGCTCGTCC
PDEB1_KpnI_fw	CGAATTGGTACCAGATAAACCGTTGGATGTC
PDEB1_BamHI_rev	GCAACTGGATCCACGAGTACTGCTGTTGTTG
p3074_Calp1.3_fw	GGTTGCATTTAAATGGCCTCGAAGAGAATGAAA
p3074_Calp1.3_rev	TCCTCCGGATCCCTTTTTTTTATTTTCCACA
p3074_CARP3_up_SwaI	CGTAGTATTTAAATGTGACATGT
p3074_CARP3_low_BamHI	CGGTATGGATCCAGAACCGTTCAATTGG
MG30	CTAGTCTAGAGGAGGAGGTTCATCCGTT

MG31	AGTATGCGGCCGCGCCATCTTCTCCTGCTCC
MG32	AGTATGCGGCCGCGAAAATAGAGAAGCGGGT
MG33	CTAGGGATCCGATTGGCGATTAAGGATG
Tb927.7.5340F10His	ATACCATGGGCCACCACCACCACCACCACCACCACCACCACGGCGCGGGCGG AGGAGGTTCATCCGTTGAAGAC
Tb927.7.5340_BamHI _rev	AATTCGGATCCTGGCTTTAGTTCAATTGGTCATCAAACAT
ESAG4_fwd_BstBI	GCAAATAAACTTTCGAAGAATGGTTGT
ESAG4-2Ty1_rev_BamHI	TCTAGTGGATCCTTAGTCAAGTGGGTCCTGGTTAGTATGGACCTCACTTGGGTC AAGTGGGTCCTGGTTAGTATGGACCTCGGTGGTAGCGCTG
CARP3_rev_ApaI	AGTGCGGGCCCTTAGTTCAATTGGTC
CARP3_NoSTOP_Spe I	TCGCACTAGTGTTCAATTGGTCATCAAAC
DCAT_Mut1*	caaaggtacgaccaggcacTGCAAGATTGTTATCACGGGTGTTGC
DCAT_Mut2*	gtgcctggtcgtacctttgCTGGTC

restriction sites are underlined

*19 bp overlaps are in lower case

Supplementary Methods

In vitro differentiation by RBP6 overexpression

Differentiation of procyclic forms by overexpression of RBP6 was performed in EATRO 1125 T7T as described previously[1,2].

Cloning and generation of transgenic trypanosomes

Generation of a homozygous carp3 knock out:

Both *CARP3* alleles were deleted from AnTat 1.1 bloodstream forms or EATRO1125 procyclic forms carrying the RBP6 overexpression construct by transfection with pTBT-based plasmids[3] containing *CARP3* 5'UTR and 3'UTR sequences flanking a hygromycin or blasticidin resistance cassette (kindly provided by Daniel Tagoe and Harry de Koning, Glasgow). Plasmids were digested with NotI and XhoI for transfection according to standard electroporation conditions[4] and cells were selected with 2 μg/mL hygromycin B or 2 μg/mL blasticidin, respectively.

Generation of endogenous CARP3 rescue and CARP3Δ3 rescue cell lines:

An endogenous CARP3 rescue cell line was generated by transfection of the hemizygous *carp3* KO cells resistant to hygromycin with a pEnT6B-based plasmid[5] that contained the *CARP3* 5'UTR amplified with primers MG30 and MG31 from genomic DNA as well as the first 300 nucleotides of the *CARP3* ORF amplified with primers MG32 and MG33. The plasmid was linearized with NotI for transfection and cells were selected with 2 μg/mL blasticidin. The same strategy was used for replacement of endogenous CARP3 by CARP3Δ3 using forward primer

CARP3Δ3_HindIII_fw instead of MG30 for amplification of the CARP3 ORF N-terminus with deletion of nucleotides 4-12.

Generation of a homozygous flam8 knock outs and rescue:

In AnTat 1.1E 'Paris' bloodstream forms, one *flam8* allele was deleted and the second one truncated to generate *flam8* knock-outs sub-clones as described in[6]. A rescue sequence was then re-introduced in the partially deleted allele to produce an add-back strain[6].

Tetracycline-inducible RNAi of CARP3:

Two copies of a tetracycline repressor were integrated into the *T. brucei* AnTat 1.1 'Munich' genome by transfection with the NotI-linearized plasmid pHD1313[7]. Antibiotic selection was performed with 10 μg/mL phleomycin. This cell line was further transfected with pHD615[PAC]CARP3_RNAi, a plasmid allowing hairpin RNAi-mediated repression of CARP3. Two fragments of the CARP3 ORF (nt 514 to 913; nt 514 to 965) were PCR-amplified from AnTat 1.1 genomic DNA using primers CARP3_hairpin_up_HindIII and CARP3_hairpin_sense_XhoI or CARP3_hairpin_up_BamHI and CARP3_hairpin_antisense_XhoI, respectively, and cloned into pHD615[PAC] by a three-component-ligation. Cells transfected with the NcoI-linearized plasmid were selected with 0.1 μg/mL puromycin.

For generation of a tetracycline-inducible CARP3 RNAi cell line in procyclic Lister 427 29-13, the plasmid p2t7-177[BLE]-CARP3 (Gould et al 2013) was used for transfection and selection was carried out with 2.5 μg/mL phleomycin.

C-terminal in situ tagging of CARP3 with YFP or mNeonGreen:

The *CARP3* C-terminus was amplified from genomic DNA (strain AnTat 1.1) using primers p3074_CARP3_up_SwaI and p3074_CARP3_low_BamHI and cloned into

the vector p3074[5] that enables C-terminal Ty1-tagging. The 4x Ty1-tag was swapped to YFP from p3329 using BamHI and EcoRI. For PALM microscopy, YFP from p3329 was replaced by mNeonGreen amplified from plasmid pK19msB-mNeonGreen-ptsG[8] by primers mNeon_BamHI_fw and mNeon_EcoRI_rev. The plasmids were linearized with NotI and transfected cells were grown in the presence of 2 μg/mL G418 or 0.1 μg/mL puromycin.

C-terminal in situ tagging of CARP3 with mCherry or photoactivatable (PA)mCherry:

For C-terminal fusion of CARP3 to mCherry, the long primer PCR tagging strategy was used[9]. YFP-Ty1 of plasmid pPOTv4 was replaced by mCherry-Ty1 followed by PCR amplification of mCherry-TY with primers pPOTv4_Lr_mCherry_TY_CARP3 fw and pPOTv4_Lr_mCherry_TY_CARP3 rev introducing stretches homologous to the C-terminus of the *CARP3* ORF and the start of the *CARP3* 3'UTR. The PCR product was purified by phenol-chloroform extraction prior to transfection and selection was done with 2 μg/mL G418. For C-terminal fusion of CARP3 to photoactivatable (PA)mCherry, the same strategy was used. mCherry-Ty1 was replaced by PAmCherry amplified from plasmid pK19mobsacBparB-PAmCherry[10] using primers PAmCherry BamHI FWD and PAmCherry SacI REV.

Overexpression of C-terminally Ty1-tagged CARP3 or CARP3 truncations.

C-terminal TY-tag fusions of full-length CARP3, CARP3 N-terminus (1-337) or CARP3 C-terminus (171-end) were generated by PCR on genomic DNA of *T. brucei* AnTat 1.1 using primers CARP3_fw_HindIII or CARP3_Ct_fw_HindIII and CARP3_Ty1_Ct_rev_BamHI or CARP3_Ty1_Nt_rev_BamHI, respectively, followed by ligation via BamHI/HindIII into the pTSARib overexpression vector[11] with a

puromycin resistance cassette[12]. Constructs were transfected into AnTat 1.1 CARP3 knockout cells and selected with 0.1 μg/mL puromycin.

Inducible expression of CARP3-BirA-Ty1 for proximity proteomics.*

Primers CARP3_fw_HindIII and CARP3_NoSTOP_SpeI were used to amplify the CARP3 ORF that was inserted together with a downstream BirA*-Ty1 cassette via HindIII, SpeI and BamHI restriction sites into plew100v5b1d-BLE, a modified version of the original plew100 vector[13].

C-terminal in situ tagging of FLAM8 with YFP or mNeonGreen:

For C-terminal in situ tagging of FLAM8 with YFP, *T. brucei* AnTat 1.1 BSFs were transfected with p3329.FLAM8[14] followed by selection with 0.1 μg/mL puromycin. For PALM microscopy, YFP was swapped to mNeonGreen using BamHI and EcoRI. The plasmids were linearized with NruI and transfected cells were grown in the presence of 0.1 μg/mL puromycin.

C-terminal in situ tagging of calpain 1.3 with mNeonGreen:

For C-terminal in situ tagging of calpain 1.3 with mNeonGreen, the *calpain 1.3* C-terminus was amplified from genomic DNA using primers p3074_Calp1.3_fw and p3074_Calp1.3_rev and cloned into p3329.mNeonGreen via KpnI and BamHI. The plasmid was linearized with NruI and transfected cells were selected with 0.1 μg/mL puromycin.

C-terminal in situ tagging of PDEB1 with mNeonGreen:

For C-terminal in situ tagging of PDEB1 with mNeonGreen, the *PDEB1* C-terminus was amplified from genomic DNA using primers PDEB1_KpnI_fw and PDEB1_BamHI_rev and cloned into p3329.mNeonGreen via KpnI and BamHI. The

plasmid was partially digested with NdeI and transfected cells were selected with 0.1 μg/mL puromycin.

<u>C-terminal in situ tagging of ACP1, ACP3, ACP4, ACP5 or ACP6 with Ty1 or mNeonGreen:</u>

C-terminal Ty1 tagging of ACP1 or ACP6, respectively, was performed similar to previously described by Saada et al.[15] using the in situ tagging vector pMO2T[16] and primers ACP1orfF/ACP1orfR and ACP1utrF/ACP1utrR or ACP6orfF/ACP6orfR and ACP6utrF2/ACP6utrR2, respectively. The Ty1 tag was swapped for mNeonGreen in plasmid pMO2T_ACP1 using primers mNeon_XhoI_fw and mNeon_SalI_rev and XhoI/SalI digestion. ACP1 ORF and 3'UTR fragments were replaced by KpnI/XhoI or BamHI/XbaI swap, respectively, with ACP3, ACP4 or ACP5 ORF and 3'UTR fragments using primers ACP3_, ACP4_ or ACP5_ORF_FW/REV and UTR_FW/REV, respectively, resulting in plasmids pMO2mNG_ACP3, pMO2mNG_ACP4 and pMO2mNG_ACP5. The plasmids were digested with EcoRV and NsiI (pMO2mNG_ACP3), KpnI and PstI (pMO2mNG_ACP4) or KpnI and SphI for transfection of procyclic AnTat 1.1 or 427 29-13 cells and selection was carried out with 1 μg/mL puromycin.

<u>Tetracycline-inducible overexpression of ESAG4ΔCAT with C-terminal GFP tag</u>

The plasmid plew82.ESAG4ΔCAT-2Ty1 (deletion of nucleotides 2680 to 3318 of ESAG4 ORF) was generated using the In-Fusion® HD Cloning Kit (Takara), Phusion® High-Fidelity DNA Polymerase (NEB) and primers DCAT_Mut1 and DCAT_Mut2 oriented in opposite directions with 19 bp overlaps on the template vector plew82.ESAG4-2Ty1. The linear plasmid was circularized in an In-Fusion reaction according to the manufacturer's instructions. In order to obtain

plew82.ESAG4ΔCAT-GFP, a 733 bp KpnI/BamHI fragment (containing EGFP) from plew82.ESAG3-DNi-2[17] was inserted into the KpnI/BamHI digested plew82.ESAG4ΔCAT-2Ty1. Linearization was done with NotI and transfected cells were selected with 2.5 μg/mL phleomycin.

Constitutive overexpression of catalytically inactive ESAG4-GFP.

Catalytically inactive ESAG4 with point mutations D948A and R1052A fused to a C-terminal GFP (ESAG4 DNi-3 from[17]) was cloned into the constitutive expression vector pTSARib[11] via HindIII and BamHI restriction sites. The plasmid was linearized with SphI and transfected into BSFs of *T. brucei* strain MiTat 1.2 13-90 CARP3 RNAi[18].

Constitutive overexpression of PKAR-GFP.

The N-terminus of *T. brucei* PKAR (protein kinase A regulatory subunit; Tb927.11.4610, amino acids 1-200) was fused to C-terminal GFP and cloned into the constitutive expression vector pTSARib[11] via a 3-component ligation using HindIII and EagI (PKAR fragment) and EagI and BamHI (GFP) restriction sites. Linearization was done with SphI and transfected cells were selected with 2 μg/mL hygromycin B.

Plasmids for expression of CARP3 and ESAG4-2Ty1 in HEK 293 cells.

The ESAG4 C-terminus (nt 2424 to 3804) was amplified from a plasmid containing the full ESAG4 (BES1/TAR40.13) ORF using primers ESAG4_fwd_BstBI and ESAG4-2Ty1_rev_BamHI introducing a C-terminal 2xTy1 tag. The fragment was ligated into plew82.ESAG4-DNi-1[17] cut with BstBI and BamHI, resulting in plasmid plew82.ESAG4-2Ty1. ESAG4-2Ty1 was cut out with BamHI and HindIII and cloned into pcDNA3.1(+).

The CARP3 ORF was amplified from plasmid pTSARib.CARP3-Ty1 using primers CARP3_fw_HindIII and CARP3_rev_ApaI and cloned into pcDNA3.1(+) via HindIII and ApaI.

A list of all primers used in this study is provided as Supplementary Table 2. All transfections were done in slender bloodstream forms, except for tagging of ACPs.

Dual-luciferase® reporter assay in HEK cAMP reporter cells.

Human embryonic kidney cells 293T (HEK 293T) were cultured in DMEM/F12 medium (Lonza) supplemented with 10% heat-inactivated FBS, 100 U/mL penicillin and 100 μg/mL streptomycin in a 5% CO_2 humidified incubator at 37°C. 2×10^5 HEK 293T cells were plated into 12-well plates and transfected after 24 h in triplicate with the plasmids pGL4.29[luc2P/CRE/Hygro] (Promega), pRL-SV40 (Renilla Luciferase), pcDNA3.1(+)ESAG4-2Ty and pcDNA3.1(+)CARP3 using Lipofectamine 2000 (Invitrogen) according to the manufacturer's instructions. The amount of plasmid DNA transfected per well was optimized for each expression vector as follows: Renilla luciferase (5 ng), Luc2P (250 ng), ESAG4 (250 ng) and CARP3 (62.5 to 500 ng). For the different conditions, the amount of pcDNA3.1(+)CARP3 varied while keeping a constant concentration of pcDNA3.1(+)ESAG4-2Ty1 (ESAG4:CARP3 ratios: 1:0, 1:0.25, 1:0.5, 1:1, 1:2, 0:1). The total amount of DNA in each well was adjusted to 1 μg with the empty pcDNA3.1(+) vector. 24 hours after transfection, cells were collected, and washed 2× with PBS. 5×10^4 cells were harvested in passive lysis buffer (Promega) and the activities of the Firefly and Renilla luciferases were measured sequentially using the Dual-Luciferase® Reporter Assay System (Promega, cat. no. E1980). Luminescence measurements were performed

for 10 s at room temperature by a Centro XS3 LB 960 luminometer (Bertold Technologies, Bad Wildbad, Germany) provided with Mikrowin 2000 v.4.41 software (Mikrotek Labsis Laborsysteme GmbH). The light resulting from cleavage of the two substrates was quantified as relative light units (RLU). Firefly:Renilla ratio was calculated by dividing firefly RLU by the Renilla RLU. The assay was performed in triplicate for each sample.

The rest of the cell pellets were resuspended in Laemmli buffer (2.5×10^3 cells/μL) and used for Western blot analysis.

AC assays

AC assays were performed according to Salmon et al.[19] after mild acid (pH 5.5) treatment. Briefly, cultures of *T. brucei* MiTat 1.2 CARP3 RNAi cells were grown to a density of around 2×10^5 cells/mL, followed by RNAi induction with 1 μg/mL doxycycline for 24 h. The cells were harvested at a density of ~1×10^6 cells/mL by centrifugation, washed twice with ice-cold phosphate-buffered saline/glucose (PSG) buffer (137 mM NaCl, 2.7 mM KCl, 8.1 mM Na_2HPO_4, 1.8 mM KH_2PO_4, 1.5% glucose, pH 8.0) and after final counting resuspended in “swell dialysis” buffer of low osmotic strength (50 mM KCl, 5 mM $MgCl_2$, 1 mM glucose, 1 mM EGTA, 1X cOmplete EDTA-free protease inhibitor cocktail, 13.3 mM TES, pH 5.5) at a density of 5×10^8 cells/mL for 1 h at 4 ºC. The reaction was started by addition of 20 μL sample (1×10^7 cells) to 80 μL assay cocktail (0.5 mM cAMP, 10 mM phosphocreatine, 50 units/mL creatine kinase, 1 mM EGTA, 10 mM $MgCl_2$, 20 mM KCl, 0.5 mM ATP, 1mM glucose, 1× cOmplete EDTA-free protease inhibitor cocktail, 25 mM TES pH 5.5 and 0.8 μCi of [a-32P]-ATP at 10-40 Ci/mmol) and was

incubated for 5, 10 and 20 min at 37°C. Each reaction was stopped by adding 100 μL of stop solution (2% SDS, 40 mM ATP, 0.01 M cAMP). [^{32}P]cAMP was isolated by two-step chromatography according to Salomon et al.[20] and measured by liquid scintillation counting. Each assay was carried out in triplicate. AC activity was calculated by linear regression analysis of the rate of cAMP production.

Cell motility assay

Procyclic forms of *T. brucei* strain AnTat 1.1 were resuspended in 70% SDM79, 10% FCS, 0.6% (v/v) methylcellulose at a density of 8 × 10^6 cells/mL and placed onto a glass slide. 12 regions of each slide were imaged on a Leica DMI600B series inverted microscope (Leica Microsystems). Cells were imaged over a period of 15 seconds, yielding 31 data points for each cell. All cells which were in view for the entire 15-second measurement period were included in the analysis. At least 50 cells were included for each cell line tested. Tracking and subsequent calculation of motion metrics was performed using the MTrackJ plugin (version 1.5.1) for ImageJ[21]. Statistical significance was determined by one-way ANOVA corrected by Šidák's multiple comparisons test with an adjusted p value ≤ 0.05 considered statistically significant.

For the FLAM8 mutant panel, movies (150 frames, 50 ms exposure) were recorded in warm SDM79 medium with 1.1% methylcellulose at 1 × 10^6 cells/mL under the 10× objective of an inverted DMI4000 LEICA microscope (Leica Microsystems) coupled to a Retiga-SRV camera (QImaging). Movies were converted with the MPEG Streamclip V.1.9b3 software (Squared 5) and analyzed with the MedeaLAB CASA Tracking V.5.5 software (Medea AV GmbH), as described in Rotureau et al.[22].

Social motility assay

Agarose plates for social motility assays were prepared as described[23]. 5 × 10^5 cells of *T. brucei* strain AnTat 1.1 or AnTat 1.1E were spotted in 5 μL of SDM-79 on agarose plates within 7 days after density-dependent differentiation from bloodstream to procyclic stage.

Generation of polyclonal antibodies

The *CARP3* ORF was cloned as N-terminal His_{10} fusion into pETDuet-1 using primers Tb927.7.5340F10His and Tb927.7.5340_BamHI_rev via NcoI and BamHI restriction sites and transformed into *E. coli* Rosetta. 500 μg of His_{10}-CARP3 purified using a Ni-NTA column (Qiagen) were used for immunization of rabbits by Eurogentec, followed by further boosts with 500 μg antigen. The CARP3 antiserum was affinity-purified using His_{10}-CARP3 according to the method of Olmsted[24].

Western blot

Western blot analysis was performed as previously described[19]. Primary antibodies used are anti-CARP3 (1:500), anti-ESAG4[19], anti-PFR-A/C[25] (1:1000), anti-Ty1[26] (1:250), anti-PAD1[27] (1:1000) and anti-RBP6[1] (1:1000). Secondary antibodies used are IRDye® 800CW Goat anti-Mouse IgG Secondary Antibody and IRDye® 680LT Goat anti-Rabbit IgG Secondary Antibody (both 1:5000, LI-COR, catalog numbers 926-32210 and 926-68021).

.

Indirect immunofluorescence analysis

For microscopic analysis, trypanosomes were either spread on glass slides and fixed in methanol for 5 min at -20°C or fixed in 2% PFA for 20 min at room temperature. Cellular DNA was visualized with 4′,6-diamidino-2-phenylindole (DAPI; 1 μg/mL). Image acquisition was performed with a DeltaVision Elite widefield fluorescence microscope (GE Healthcare) equipped with a CoolSnap HQ2 CCD camera (Photometrics, Arizona, USA) using softWoRx version 6.1.1 and images were processed with Fiji/ImageJ version 2.1.0[28,29]. Primary antibodies used are anti-CARP3 (1:150), anti-Ty1[26] (1:100), anti-SAXO[30] (mAB25; 1:25), anti-EP procyclin (cedarlane, cat. no. CLP001AP, 1:500), anti-calflagin[31] (1:1000). Secondary antibodies used are Alexa Fluor™ 488 goat anti-mouse IgG (H+L) and Alexa Fluor™ 594 goat anti-rabbit IgG (H+L) (both 1:500, Invitrogen, ThermoFisher, catalog numbers A-11001 and A-11012).

Live cell fluorescence microscopy

For live cell fluorescence microscopy, procyclic trypanosomes were resuspended in PBS and imaged using a DeltaVision Elite widefield fluorescence microscope (GE Healthcare) equipped with a CoolSnap HQ2 CCD camera (Photometrics, Arizona, USA) using softWoRx version 6.1.1. Images were processed with ImageJ 2.1.0[28,29].

Pull-down with cAMP agarose

3 × 10^8 procyclic *T. brucei* cells were washed twice with PBS and lysed in lysis buffer (10 mM Tris/Cl pH 7.5; 150 mM NaCl; 0.5 mM EDTA; 0.5% NP-40; Roche cOmplete

protease inhibitor) for 30 min at 4°C. Lysates were incubated with plain agarose beads (Biolog Bremen) for 1 h at 4°C in order to remove proteins binding non-specifically to the bead matrix. Pull-downs were performed by incubation of the pre-cleared lysates with 60 μL 2-AHA- or 8-AHA-agarose (Biolog Bremen Cat. No. A054, A028) beads slurry for 2 h at 4°C, followed by five washes with lysis buffer. Bound proteins were eluted by boiling (5 min 95°) with 40 μL 2× Laemmli sample buffer.

Immunoprecipitations

Immunoprecipitation of CARP3 or of Ty1-tagged bait proteins was performed by binding anti-CARP3 or anti-Ty1 to magnetic protein A beads (Dynabeads, Invitrogen) followed by a 2-hour incubation with 1×10^8 trypanosomes lysed in lysis buffer (10 mM Tris/Cl pH 7.5; 150 mM NaCl; 0.5 mM EDTA; 0.5% NP-40; Roche cOmplete protease inhibitor) for 30 min at 4°C. Beads were washed 4x with lysis buffer and proteins were eluted by incubation with 50 μL 2× Laemmli sample buffer for 5 min at 95°C. Immunoprecipitations from HEK293T cells were carried out essentially using the same protocol with protein A sepharose beads and 5×10^5 cells.

Proximity proteomics using CARP3 BioID

BioID was adapted from[32]. Briefly, 4-5 × 10^8 *T. brucei* MiTat 1.2 BSF cells expressing tetracycline-induced (1 μg/mL, 24 h) CARP3-BirA*-Ty1 or CARP3 (control) were treated with 50 μM biotin for 24 h (n = 3 replicates). Cells were harvested by centrifugation, washed 3× with PBS and lysed in 500 μL lysis buffer (50 mM Tris/Cl

pH 7.4; 500 mM NaCl; 5 mM EDTA; 0.4% SDS; 1 mM DTT; Roche cOmplete protease inhibitor) by sonication (Bioruptor; 2 cycles with 30” on/off, high energy). Triton X-100 was added to a final concentration of 2%, followed by sonication as above. Addition of 500 μL Tris/Cl pH 7.4 was followed by another round of sonication. Soluble proteins were separated by centrifugation (15 min, 16,000 × g, 4°C) and subjected to buffer exchange using a PD-10 column (GE Healthcare) according to the manufacturer’s instructions. The eluate was concentrated (Spin-X UF 6 concentrator column) to < 1mL and incubated with 50 μL streptavidin-coupled magnetic beads (Dynabeads ® MyOne™ Streptavidin T1, ThermoFisher) overnight at 4°C on an overhead rotator. Beads were washed 5x with PBS, followed by elution with Laemmli sample buffer for 10 min at 95°C. Sample preparation for mass spectrometry, protein digestion and LC-MS parameters used for nanoLC-MS/MS analysis on a nanospray Q Exactive were previously described[12]. Raw spectra were analyzed with MaxQuant version 1.6.17.0[33], which incorporates the Andromeda search engine, using the *Trypanosoma brucei* TriTrypDB-51_TbruceiTREU927 protein database and the following settings: three missed cleavages from trypsin digestion were allowed; carbamidomethyl-cysteine was set as fixed modification and biotinylation (K), oxidation (M), acetylation (protein N-terminal) and deamidation (N, Q) were set as dynamic modifications. The MaxQuant output was loaded into Perseus version 1.6.7.0[34] and filtered to exclude proteins ‘only identified by site’, reverse hits and potential contaminants. The LFQ values of the remaining proteins were $\log_2$ transformed and missing values were replaced from normal distribution. Only proteins identified in all three pull-downs were considered for further analysis. Proteins were considered as statistically significant with FDR ≤ 0.05 and $s_0 = 2$ (two-

sided Student's T-test). The raw and processed mass spectrometry proteomics data have been deposited to the ProteomeXchange Consortium[35] (http://proteomecentral.proteomexchange.org) via the PRIDE partner repository[36] with the dataset identifier PXD025357.

Quantitative proteomics upon CARP3 knock-down

3 × 10^7 *T. brucei* MiTat 1.2 13-90 BSF cells with tetracycline-inducible *CARP3* RNAi knock-down were induced or not with 5 μg/mL tetracycline for 24 h (n = 4 replicates). Cell lysis, protein digestion, peptide purification and MS/MS analysis were performed as described by Humphrey et al.[37]. Purified peptides were injected in an RSLCnano system (Thermo) and separated in a 25-cm analytical Aurora C18 nanocolumn (75 µm ID 120 Å, 1.6 µm, Ion Opticks) with a 120-min gradient from 4 to 40% acetonitrile in 0.1% formic acid. The effluent from the HPLC was directly electrosprayed into a Q Exactive HF (Thermo), operated in data dependent mode to automatically switch between full scan MS and MS/MS acquisition. Survey full scan MS spectra (from m/z 375–1600) were acquired with resolution R = 60000 at m/z 400 (AGC target of $3x10^6$). The ten most intense peptide ions with charge states between 3 and 5 were sequentially isolated to a target value of $1x10^5$ and fragmented at 27% normalized collision energy. Typical mass spectrometric conditions were: spray voltage, 1.5 kV; no sheath and auxiliary gas flow; heated capillary temperature, 250°C; ion selection threshold, 33000 counts.

Protein identification and quantification (LFQ) was performed using MaxQuant version 1.6.10.43[33] with the following parameters: Database, TriTrypDB-46_TbruceiTREU927_AnnotatedProteins; MS tol, 10 ppm; MS/MS tol, 20 ppm;

Peptide FDR, 0.1; Protein FDR, 0.01 Min. peptide Length, 5; Variable modifications, Oxidation (M); Fixed modifications, Carbamidomethyl (C); Peptides for protein quantitation, razor and unique; Min. peptides, 1; Min. ratio count, 2. Statistical analysis was performed using Perseus 1.6.7.0[34] with the following workflow: proteins only identified by site, reverse hits or potential contaminants were filtered out. Only proteins that were identified in at least three of the four non-induced replicate samples were considered for further analysis. The LFQ values of the remaining proteins were $\log_2$ transformed and missing values were replaced from normal distribution. Statistical significance was evaluated with a two-sided Student's t-test with FDR ≤ 0.05 and $s_0 = 0.1$. The raw and processed mass spectrometry proteomics data have been deposited to the ProteomeXchange Consortium[35] (http://proteomecentral.proteomexchange.org) via the PRIDE partner repository[36] with the dataset identifier PXD025401.

Supplementary References

1 Kolev, N. G., Ramey-Butler, K., Cross, G. A. M., Ullu, E. & Tschudi, C. Developmental Progression to Infectivity in *Trypanosoma brucei* Triggered by an RNA-Binding Protein. *Science (New York, N.Y.)* **338**, 1352-1353, doi:10.1126/science.1229641 (2012).

2 Wargnies, M. *et al.* Gluconeogenesis is essential for trypanosome development in the tsetse fly vector. *PLOS Pathogens* **14**, e1007502, doi:10.1371/journal.ppat.1007502 (2018).

3 Cross, M. *et al.* J-binding protein increases the level and retention of the unusual base J in trypanosome DNA. *Molecular microbiology* **46**, 37-47 (2002).

4 Schumann Burkard, G., Jutzi, P. & Roditi, I. Genome-wide RNAi screens in bloodstream form trypanosomes identify drug transporters. *Molecular and biochemical parasitology* **175**, 91-94, doi:10.1016/j.molbiopara.2010.09.002 (2011).

5 Kelly, S. *et al.* Functional genomics in *Trypanosoma brucei*: a collection of vectors for the expression of tagged proteins from endogenous and ectopic gene loci. *Molecular and biochemical parasitology* **154**, 103-109, doi:10.1016/j.molbiopara.2007.03.012 (2007).

6 Calvo-Alvarez, E., Crouzols, A. & Rotureau, B. FLAgellum Member 8 modulates extravascular trypanosome distribution in the mammalian host. *bioRxiv*, 2021.2001.2008.425862, doi:10.1101/2021.01.08.425862 (2021).

7 Alibu, V. P., Storm, L., Haile, S., Clayton, C. & Horn, D. A doubly inducible system for RNA interference and rapid RNAi plasmid construction in *Trypanosoma brucei*. *Molecular and biochemical parasitology* **139**, 75-82, doi:10.1016/j.molbiopara.2004.10.002 (2005).

8 Martins, G., Giacomelli, G., Goldbeck, O., Seibold, G. & Bramkamp, M. Substrate-dependent cluster density dynamics of *Corynebacterium glutamicum* phosphotransferase system permeases. *Molecular microbiology* **111**, doi:10.1111/mmi.14224 (2019).

9 Dean, S. *et al.* A toolkit enabling efficient, scalable and reproducible gene tagging in trypanosomatids. *Open biology* **5**, 140197-140197, doi:10.1098/rsob.140197 (2015).

10 Böhm, K. *et al.* Chromosome organization by a conserved condensin-ParB system in the actinobacterium *Corynebacterium glutamicum*. *bioRxiv*, 649749, doi:10.1101/649749 (2019).

11 Xong, H. V. *et al.* A VSG expression site-associated gene confers resistance to human serum in *Trypanosoma rhodesiense*. *Cell* **95**, 839-846 (1998).

12 Bachmaier, S. *et al.* Nucleoside analogue activators of cyclic AMP-independent protein kinase A of *Trypanosoma*. *Nature Communications* **10**, 1421, doi:10.1038/s41467-019-09338-z (2019).

13 Wirtz, E., Leal, S., Ochatt, C. & Cross, G. A. A tightly regulated inducible expression system for conditional gene knock-outs and dominant-negative genetics in *Trypanosoma brucei*. *Molecular and biochemical parasitology* **99**, 89-101 (1999).

14 Subota, I. *et al.* Proteomic analysis of intact flagella of procyclic *Trypanosoma brucei* cells identifies novel flagellar proteins with unique sub-localization and dynamics. *Molecular & cellular proteomics : MCP* **13**, 1769-1786, doi:10.1074/mcp.M113.033357 (2014).

15 Saada, E. A. *et al.* Insect Stage-Specific Receptor Adenylate Cyclases Are Localized to Distinct Subdomains of the *Trypanosoma brucei* Flagellar Membrane. *Eukaryotic Cell* **13**, 1064-1076, doi:10.1128/EC.00019-14 (2014).

16 Oberholzer, M., Morand, S., Kunz, S. & Seebeck, T. A vector series for rapid PCR-mediated C-terminal in situ tagging of *Trypanosoma brucei* genes. *Molecular and biochemical parasitology* **145**, 117-120, doi:10.1016/j.molbiopara.2005.09.002 (2006).

17 Salmon, D. *et al.* Adenylate cyclases of *Trypanosoma brucei* inhibit the innate immune response of the host. *Science (New York, N.Y.)* **337**, 463-466, doi:10.1126/science.1222753 (2012).

18 Gould, M. K. *et al.* Cyclic AMP effectors in African trypanosomes revealed by genome-scale RNA interference library screening for resistance to the phosphodiesterase inhibitor CpdA. *Antimicrobial agents and chemotherapy* **57**, 4882-4893, doi:10.1128/AAC.00508-13 (2013).

19 Salmon, D. *et al.* Cytokinesis of *Trypanosoma brucei* bloodstream forms depends on expression of adenylyl cyclases of the ESAG4 or ESAG4-like subfamily. *Molecular microbiology* **84**, 225-242, doi:10.1111/j.1365-2958.2012.08013.x (2012).

20 Salomon, Y. Adenylate cyclase assay. *Adv Cyclic Nucleotide Res* **10**, 35-55 (1979).

21 Meijering, E., Dzyubachyk, O. & Smal, I. Methods for cell and particle tracking. *Methods in enzymology* **504**, 183-200, doi:10.1016/b978-0-12-391857-4.00009-4 (2012).

22 Rotureau, B. *et al.* Flagellar adhesion in *Trypanosoma brucei* relies on interactions between different skeletal structures in the flagellum and cell body. *Journal of cell science* **127**, 204-215, doi:10.1242/jcs.136424 (2014).

23 Oberholzer, M., Lopez, M. A., McLelland, B. T. & Hill, K. L. Social Motility in African Trypanosomes. *PLoS Pathogens* **6**, e1000739, doi:10.1371/journal.ppat.1000739 (2010).

24 Olmsted, J. B. Affinity purification of antibodies from diazotized paper blots of heterogeneous protein samples. *The Journal of biological chemistry* **256**, 11955-11957 (1981).

25 Kohl, L., Sherwin, T. & Gull, K. Assembly of the paraflagellar rod and the flagellum attachment zone complex during the *Trypanosoma brucei* cell cycle. *The Journal of eukaryotic microbiology* **46**, 105-109 (1999).

26 Bastin, P., Bagherzadeh, Z., Matthews, K. R. & Gull, K. A novel epitope tag system to study protein targeting and organelle biogenesis in *Trypanosoma brucei*. *Molecular and biochemical parasitology* **77**, 235-239 (1996).

27 Dean, S., Marchetti, R., Kirk, K. & Matthews, K. R. A surface transporter family conveys the trypanosome differentiation signal. *Nature* **459**, 213-217, doi:10.1038/nature07997 (2009).

28 Schindelin, J. *et al.* Fiji: an open-source platform for biological-image analysis. *Nature methods* **9**, 676-682, doi:10.1038/nmeth.2019 (2012).

29 Schneider, C. A., Rasband, W. S. & Eliceiri, K. W. NIH Image to ImageJ: 25 years of image analysis. *Nature methods* **9**, 671-675 (2012).

30 Dacheux, D. *et al.* A MAP6-related protein is present in protozoa and is involved in flagellum motility. *PloS one* **7**, e31344-e31344, doi:10.1371/journal.pone.0031344 (2012).

31 Giroud, C. *et al.* Murine Models for *Trypanosoma brucei* gambiense disease progression--from silent to chronic infections and early brain tropism. *PLoS Negl Trop Dis* **3**, e509-e509, doi:10.1371/journal.pntd.0000509 (2009).

32 Morriswood, B. *et al.* Novel bilobe components in *Trypanosoma brucei* identified using proximity-dependent biotinylation. *Eukaryot Cell* **12**, 356-367, doi:10.1128/ec.00326-12 (2013).

33 Cox, J. & Mann, M. MaxQuant enables high peptide identification rates, individualized p.p.b.-range mass accuracies and proteome-wide protein quantification. *Nature biotechnology* **26**, 1367-1372, doi:10.1038/nbt.1511 (2008).

34 Tyanova, S., Temu, T. & Sinitcyn, P. The Perseus computational platform for comprehensive analysis of (prote)omics data. **13**, 731-740, doi:10.1038/nmeth.3901 (2016).

35 Vizcaíno, J. A. *et al.* ProteomeXchange provides globally coordinated proteomics data submission and dissemination. *Nature biotechnology* **32**, 223-226, doi:10.1038/nbt.2839 (2014).

36 Vizcaíno, J. A. *et al.* 2016 update of the PRIDE database and its related tools. *Nucleic Acids Research* **44**, D447-D456, doi:10.1093/nar/gkv1145 %J Nucleic Acids Research (2015).

37 Humphrey, S. J., Karayel, O., James, D. E. & Mann, M. High-throughput and high-sensitivity phosphoproteomics with the EasyPhos platform. *Nature protocols* **13**, 1897-1916, doi:10.1038/s41596-018-0014-9 (2018).
