# Supplementary material for: A multi-adenylate cyclase regulator at the flagellar tip controls African trypanosome transmission
Source: Nat Commun. 2022 Sep 16;13:5445. doi: 10.1038/s41467-022-33108-z (PMC9481589; doi:10.1038/s41467-022-33108-z)
Supplement: Supplementary file 3 — Description of Additional Supplementary Files [file 41467_2022_33108_MOESM3_ESM.docx]

**Description of additional supplementary files**

Bachmaier et al., A multi-adenylate cyclase regulator at the flagellar tip controls

African trypanosome transmission

File title: Supplementary Data 1

Description: CARP3-YFP pull-down in bloodstream (A) and procyclic forms (B).

File title: Supplementary Data 2

Description: ESAG4-GFP pull-down in bloodstream forms.

File title: Supplementary Data 3

Description: CARP3 BioID proximity proteomics identifies putative CARP3 interactors.

File title: Supplementary Data 4

Description: Quantitative proteomics upon CARP3 knock down.

File title: Supplementary Data 5

Description: Summary of the *T. brucei* AC repertoire.

File title: Supplementary Data 6

Description: Pymol states of seven AlphaFold-generated models of CARP3 in complex with the intracellular catalytic domain of different ACs, corresponds to Supplementary Fig. 9a.

File title: Supplementary Data 7

Description: Pymol states of an AlphaFold-generated model for an ESAG4 AC homodimer, corresponds to Supplementary Fig. 9d.
