# Supplementary material for: A multi-adenylate cyclase regulator at the flagellar tip controls African trypanosome transmission
Source: Nat Commun. 2022 Sep 16;13:5445. doi: 10.1038/s41467-022-33108-z (PMC9481589; doi:10.1038/s41467-022-33108-z)
Supplement: Supplementary file 11 — Reporting Summary [file 41467_2022_33108_MOESM11_ESM.pdf]

nature portfolio

Corresponding author(s): Bachmaier, S., Boshart, M.

Last updated by author(s): Aug 24, 2022

Reporting Summary

Nature Portfolio wishes to improve the reproducibility of the work that we publish. This form provides structure for consistency and transparency in reporting. For further information on Nature Portfolio policies, see our Editorial Policies and the Editorial Policy Checklist.

Statistics

For all statistical analyses, confirm that the following items are present in the figure legend, table legend, main text, or Methods section.

n/a	Confirmed	
☐	☒	The exact sample size (*n*) for each experimental group/condition, given as a discrete number and unit of measurement
☐	☒	A statement on whether measurements were taken from distinct samples or whether the same sample was measured repeatedly
☐	☒	The statistical test(s) used AND whether they are one- or two-sided<br>*Only common tests should be described solely by name; describe more complex techniques in the Methods section.*
☒	☐	A description of all covariates tested
☐	☒	A description of any assumptions or corrections, such as tests of normality and adjustment for multiple comparisons
☐	☒	A full description of the statistical parameters including central tendency (e.g. means) or other basic estimates (e.g. regression coefficient) AND variation (e.g. standard deviation) or associated estimates of uncertainty (e.g. confidence intervals)
☐	☒	For null hypothesis testing, the test statistic (e.g. *F*, *t*, *r*) with confidence intervals, effect sizes, degrees of freedom and *P* value noted<br>*Give P values as exact values whenever suitable.*
☒	☐	For Bayesian analysis, information on the choice of priors and Markov chain Monte Carlo settings
☒	☐	For hierarchical and complex designs, identification of the appropriate level for tests and full reporting of outcomes
☒	☐	Estimates of effect sizes (e.g. Cohen's *d*, Pearson's *r*), indicating how they were calculated

Our web collection on statistics for biologists contains articles on many of the points above.

Software and code

Policy information about availability of computer code

Data collection	Peak fit, lateral drift and channel alignment for PALM microscopy were performed with ZEN Black (ZEN Black 2.1 SP3, version 14.0.4.201, 64-bit).<br><br>Movies of live trypanosomes were converted with the MPEG Streamclip V.1.9b3 software (Squared 5). Single cell motility tracking was performed using the MTrackJ plugin (version 1.5.1) for ImageJ version 2.1.0 or the MedeaLAB CASA Tracking V.5.5 software (Medea AV GmbH).<br><br>Fluorescence microscopy image acquisition was carried out with Micro-Manager 1.4 or softWoRx Version 6.1.1 (Applied Precision).<br><br>For mass spectrometry searches, the trypanosome protein databases Trypanosoma brucei TriTrypDB-42_TbruceiTREU927_AnnotatedProteins, TriTrypDB-46_TbruceiTREU927_AnnotatedProteins, TriTrypDB-51_TbruceiTREU927_AnnotatedProteins, or TriTrypDB-48_TbruceiLISTER427_AnnotatedProteins, and MaxQuant versions 1.6.3.4, 1.6.10.43, 1.6.14.0, or 1.6.17.0 were used.
Data analysis	Localization and colocalization analysis for PALM microscopy was performed via in house Fiji version 2.1.0 and R scripts (http://www.R-project.org/) (https://github.com/GiacomoGiacomelli/Carp3-Co-localization-PALM), written in RStudio (version 1.1.456) (RStudio_Team 2016) and run on R version 3.6.3 (2020-02-29) (R_Core_Team 2020).<br><br>For microscopy analyses, Fiji/Image versions 1.8.0 - 2.1.0/1.53c were used.

For statistical analyses of mass spectrometry datasets, Perseus version 1.6.7.0 was used.

For manuscripts utilizing custom algorithms or software that are central to the research but not yet described in published literature, software must be made available to editors and reviewers. We strongly encourage code deposition in a community repository (e.g. GitHub). See the Nature Portfolio guidelines for submitting code & software for further information.

Data

Policy information about availability of data

All manuscripts must include a data availability statement. This statement should provide the following information, where applicable:

- Accession codes, unique identifiers, or web links for publicly available datasets
- A description of any restrictions on data availability
- For clinical datasets or third party data, please ensure that the statement adheres to our policy

The proteomics datasets are available in the PRIDE partner repository with the dataset identifiers PXD025398 (CARP3-YFP pull down), PXD025412 (ESAG4-GFP pull down), PXD025357 (CARP3 BioID) and PXD025401 (CARP3 RNAi quantitative proteomics). Genome sequence and annotation information for CARP3 (Tb927.7.5340) and AC isoforms ESAG4 (Tb427.BES40.13), ACP1 (Tb927.11.17040), ACP3 (Tb927.7.7470), ACP4 (Tb927.10.13040), ACP5 (Tb927.11.13740), ACP6 (Tb927.9.15660), GRESAG4.1 (Tb927.6.760) and BSAL_05460 and for mass spectrometry data was obtained from TriTrypDB (https://tritrypdb.org).
The source data underlying Fig. 1a, b, d, Fig. 2b, Fig. 4a-f, Fig. 5c, Fig. 6a, b, d, e, and Supplementary Fig. 1b-g, Supplementary Fig. 4b, Supplementary Fig 5b, Supplementary Fig. 6a-c, Supplementary Fig. 7a, Supplementary Fig. 8g,h are provided as source data file.

Human research participants

Policy information about studies involving human research participants and Sex and Gender in Research.

Reporting on sex and gender	n/a
Population characteristics	n/a
Recruitment	n/a
Ethics oversight	n/a

Note that full information on the approval of the study protocol must also be provided in the manuscript.

Field-specific reporting

Please select the one below that is the best fit for your research. If you are not sure, read the appropriate sections before making your selection.

☒ Life sciences ☐ Behavioural & social sciences ☐ Ecological, evolutionary & environmental sciences

For a reference copy of the document with all sections, see nature.com/documents/nr-reporting-summary-flat.pdf

Life sciences study design

All studies must disclose on these points even when the disclosure is negative.

Sample size	The number of experimental replicates was determined according to experience (following GLP) to ascertain reproducibility and statistical significance. Tsetse fly experiments are limited by resources, hence samples sizes chosen were limited by availability of flies.
Data exclusions	No data were excluded.
Replication	See new section 'statistics and reproducibility' in Methods.
Randomization	Treatments/inductions were done on a homogeneous starting population (flies, cells) at the same time in parallel.
Blinding	Tsetse infections and dissections were performed by the same investigator.

Reporting for specific materials, systems and methods

We require information from authors about some types of materials, experimental systems and methods used in many studies. Here, indicate whether each material, system or method listed is relevant to your study. If you are not sure if a list item applies to your research, read the appropriate section before selecting a response.

Materials & experimental systems

n/a	Involved in the study
	☒ Antibodies
	☒ Eukaryotic cell lines
☒	Palaeontology and archaeology
	☒ Animals and other organisms
☒	Clinical data
☒	Dual use research of concern

Methods

n/a	Involved in the study
☒	ChIP-seq
☒	Flow cytometry
☒	MRI-based neuroimaging

Antibodies

Antibodies used

anti-CARP3: polyclonal antibody raised in rabbits against recombinant T. brucei CARP3, described in this study

anti-ESAG4: polyclonal rabbit antibody detecting a C-terminal peptide of T. brucei ESAG4; described by Salmon et al. 2012, Mol Microbiol 84, 225-242; provided by D. Salmon, coauthor on the current manuscript

anti-PFR-A/C: monoclonal mouse antibody, clone L13D6, detecting T. brucei PFR-A/C; described by Kohl et al. 1999, J. Euk Microbiol 46, 105ff; provided by L. Kohl and P. Bastin

anti-Ty1: monoclonal mouse antibody, clone BB2 (mouse IgG1), detecting the 10-amino acid Ty1 epitope tag; described by Bastin et al. 1996, Mol Biochem Parasitol. 77, 235ff; provided by K. Gull

anti-PAD1: polyclonal rabbit antibody raised against two peptides of T. brucei PAD1; described by Dean et al. 2009, Nature 459, 213-217; provided by K. Matthews

anti-RBP6: polyclonal rabbit antibody detecting an N-terminal peptide of T. brucei RBP6; described by Kolev et al. 2012, Science 338, 1352-1353; provided by F. Bringaud

anti-SAXO: monoclonal mouse antibody, clone mAB25 (mouse IgG2a), detecting T. brucei SAXO; described by Dacheux et al. 2012, PloS one 7, e31344-e31344; provided by M. Bonhivers

anti-EP procyclin: commercial, Cedarlane, Cat. No. CLP001AP, Clone TBRP1/247, purified, mouse IgG1, lot P115

anti-calflagin: monoclonal mouse antibody detecting T. brucei calflagin; described by Giroud et al. 2009, PLoS Negl Trop Dis 3, e509-e509, provided by F. Bringaud

anti-FLAM8: polyclonal rabbit antibody detecting T. brucei FLAM8, described by Bertiaux et al., 2018, Current Biology 28, 3802-3814.e3803, original source Paul McKean, University of Lancaster, UK, provided by B. Rotureau, coauthor on the current manuscript

Alexa Fluor® 488 AffiniPure Goat Anti-Rabbit IgG (H+L), Jackson ImmunoResearch, Catalog No. 111-545-003

Cy™5 AffiniPure Goat Anti-Rabbit IgG (H+L), Jackson ImmunoResearch, Catalog No. 111-175-144,

IRDye® 800CW Goat anti-Mouse IgG Secondary Antibody, catalog no. 926-32210, lot D10128-15

IRDye® 680LT Goat anti-Rabbit IgG Secondary Antibody, catalog no. 926-68021, lot C90910

Validation

anti-CARP3: validated in this study for specific detection of T. brucei CARP3 by Western blot using wild type, carp3 KO and knock down cell lines (Fig. 1a, b); validated by immunofluorescence using wild type and carp3 KO cell lines (Fig. 1e, f).

anti-ESAG4: validated for specific detection of T. brucei ESAG4 by Western blot using wild type and esag4 KO cell lines by Salmon et al. 2012, Mol Microbiol 84, 225-242

anti-PFR-A/C: validated for specific detection of T. brucei PFR-A/C by Western blot and immunofluorescence by Kohl et al. 1999, J. Euk Microbiol 46, 105ff

anti-Ty1: validated for specific detection of Ty1-tagged proteins in T. brucei by Bastin et al., 1996, Mol Biochem Parasitol. 77, 235ff

anti-PAD1: validated for specific detection of the T. brucei stumpy bloodstream form marker protein PAD1 by Western blot and immunofluorescence of pleomorphic T. brucei AnTat 1.1 cells by Dean et al. 2009, Nature 459, 213-217.

anti-RBP6: validated for specific detection of T. brucei RBP6 by Western blot in tetracycline-inducible RBP6 overexpressing T. brucei cells by Kolev et al. 2012, Science 338, 1352-1353.

anti-SAXO: validated for specific detection of the axoneme-associated protein SAXO in T. brucei by Western blot detection of SAXO in wild type and SAXO-GFP overexpressing T. brucei cells and of recombinantly expressed SAXO.

anti-EP procyclin: species- and stage-specific surface binding of this monoclonal mouse antibody, clone TBRP1/247, was shown by immunofluorescence microscopy on living and fixed T. brucei cells (procyclic vs. long slender and short stumpy bloodstream form) and by Western blot detection of procyclic/promastigote lysates of T. brucei vs. T. congolense and Leishmania by Richardson et al. 1986 J. Immunol. 136, 2259-2264 and Richardson et al. 1988, Mol Biochem Parasitol 31, 203-216. EP peptide binding specificity was shown by indirect ELISA.

anti-calflagin: validated for specific detection of T. brucei calflagin by Western blot detection by Giroud et al. 2009, PLoS Negl Trop Dis 3, e509-e509.

anti-FLAM8: validated for specific detection of T. brucei FLAM8 by immunofluorescence analysis on fixed T. brucei cells by Bertiaux et al., 2018, Current Biology 28, 3802-3814.e3803 and Calvo-Alvarez et al., 2021 bioRxiv, 2021.2001.2008.425862.

Eukaryotic cell lines

Policy information about cell lines and Sex and Gender in Research

Cell line source(s)	All T. brucei brucei cell lines are derived in the laboratory either from T. brucei brucei stock Lister 427 clone MiTat 1.2, originally obtained from G. Cross, NY (cited in Methods), or from AnTat 1.1, as described in Bachmaier et al. 2020 MiMB 2116, 23-38. The T. brucei strain AnTat 1.1 Munich was originally obtained from the Institute of Tropical Medicine Antwerp via E. Pays and P. Overath.<br>HEK293T cells were obtained from Benoit Vanhollebeke and David Pérez Morga (ULB, IBBM), originally commercially obtained from ATCC (CRL-3216).
Authentication	T. brucei brucei stock Lister 427 clone MiTat 1.2 was verified by staining with the VSG 221 antibody. The pleomorphic properties of T. brucei brucei AnTat 1.1 Munich were regularly verified by matrix-dependent growth and differentiation assays. No authentication procedure was used for HEK293T cells.
Mycoplasma contamination	HEK293T cells were monthly tested negative for mycoplasma contamination.
Commonly misidentified lines<br>(See ICLAC register)	Only HEK293 cells and trypanosome strains were used. No commonly misidentified lines were used.

Animals and other research organisms

Policy information about studies involving animals; ARRIVE guidelines recommended for reporting animal research, and Sex and Gender in Research

Laboratory animals	Female OF1 mice of 10-14 weeks were used for trypanosome infection and were housed in stable groups of compatible individuals at medium-density caged with max. 5 mice per cage. The animals' environment was kept within specifically defined limits: temperature of 19.5-24.5°C and relative humidity of 45-65%. A 12h day/ 12h night cycle was respected. Solid food pellets were given to motivate chewing behavior and to maintain the teeth. To allow the animals to express their natural behavior as best as possible, all animals were provided with cage enrichment like a red mouse-retreat, Nestlets®, a cardboard tunnel and extra paper.<br><br>Tsetse flies (Glossina morsitans morsitans) were maintained at the Institute of Tropical Medicine, Antwerp, or at the Trypanosome Transmission Group's insectarium of the Institut Pasteur at 27°C with 70% relative humidity in Roubaud cages. Teneral males were used for all experiments = unfed adults emerged from their puparium since 12 h to 72 h
Wild animals	the study did not involve wild animals
Reporting on sex	sex was not considered in the study design
Field-collected samples	the study did not involve samples collected from the field
Ethics oversight	ITM Animal Ethics Committee clearance nr. VPU2014-1

Note that full information on the approval of the study protocol must also be provided in the manuscript.
